# Microfluidic Devices for Manufacture of Therapeutic Extracellular Vesicles: Advances and Opportunities

**DOI:** 10.1002/jev2.70132

**Published:** 2025-07-24

**Authors:** Amin Hassanzadeh‐Barforoushi, Xenia Sango, Ella L. Johnston, David Haylock, Yuling Wang

**Affiliations:** ^1^ School of Natural Sciences, Faculty of Science and Engineering Macquarie University Sydney NSW Australia; ^2^ VivaZome Therapeutics Pty Ltd Melbourne VIC Australia

## Abstract

Extracellular vesicles (EVs) are emerging as promising candidates in therapeutic applications due to their unique ability to mediate intercellular communication and deliver biological cargo. With increasing interest in EV‐based therapies, the development of scalable, cost‐effective and regulatory‐compliant production methods is critical. Microfluidic platforms offer transformative potential in EV manufacturing, providing precise control over production conditions, enhanced purity and seamless integration with quality control systems. This review highlights the advantages of microfluidic technologies in EV production, including fine‐tuning of shear stress to optimise yield, advanced purification strategies that achieve high recovery and purity, and on‐chip capabilities for EV loading and surface modification. Key challenges such as scaling up production while maintaining sterility, controlling EV release after immunoaffinity capture, and addressing clogging and fouling in microfluidic devices are discussed alongside emerging solutions. Additionally, the integration of AI‐driven automation and real‐time monitoring, as well as personalised EV manufacturing, is explored as pivotal innovations. Future directions emphasise the potential of combining size‐ and affinity‐based methods for EV isolation and aligning microfluidic technologies with regulatory requirements to accelerate clinical translation. Therefore, we believe microfluidics platforms for EV isolation hold immense potential to redefine EV manufacturing by enabling scalable, reproducible and high‐quality production systems essential for therapeutic applications.

## 1 Introduction

Extracellular vesicles (EVs), delimited by a lipid bilayer and ranging from 30 nm–1 µm in size, released from eukaryotic and prokaryotic cells, play important roles in the removal of cell waste products and cell–cell communication (EL Andaloussi et al. 2013; Zaborowski et al. 2015; György et al. 2011). During the last few decades there has been a burgeoning body of published information describing the nature and function of EVs (Théry et al. 2018; Spada and Galluzzi 2020; Doyle and Wang 2019). It is now well recognised that cells produce a range of extracellular particles, such as exomeres, small EVs (sEV), apoptotic bodies and oncosomes where the characteristics and function of each particle type are uniquely linked to their parental cell, and dependent on their mode of biogenesis (Willms et al. 2016; Crescitelli et al. 2021). Due to their abundance, biological properties and therapeutic potential, in this review, we focus on 50–150 nm bilipid layer bound sEV (Huotari and Helenius 2011; Hessvik and Llorente 2018). The luminal biological cargo, lipid bilayer composition, including lipids and lipid‐spanning proteins of sEV vary with different cells (Willms et al. 2016; Greening et al. 2017; Stępień et al. 2021). The ability of sEV to carry and deliver a mixture of biological cargo, including nucleic acids (micro‐RNAs and messenger RNA), proteins and lipids, makes them a potent intercellular communication system that regulates the fate and function of nearby or distant cells. As such, sEV play important roles in normal physiological processes and disease (Cheng et al. 2018; Salomon and Rice 2017; Tian et al. 2019; Roma‐Rodrigues et al. 2014).

Although lipid nanoparticles (LNPs) have been widely employed as delivery vehicles due to their formulation simplicity and scalable production (Xu et al. 2022), EVs offer distinct biological advantages (Zhang et al. 2023). EVs naturally exhibit reduced immunogenicity and toxicity, possess superior endosomal escape capabilities and maintain complex membrane protein structures that are challenging to replicate in synthetic systems (Murphy et al. 2021; Santos and Almeida 2021). Furthermore, EVs can be engineered for targeted delivery and exhibit natural targeting properties based on the characteristics of their source cells.

The increased interest and emerging investment in EV‐based therapeutics are underpinned by the ability of EVs to deliver multiple biological cargo to a broad range of organs, tissues and specific cell types. Indeed, there has been a significant increase in the number of EV‐based clinical therapy trials during the last 7 years (Mizenko et al. 2024). Notably, 61 individual diseases have been treated using EV therapies, with a particular focus on the treatment of acute and long forms of COVID‐19, acute respiratory distress syndrome and other respiratory illnesses.

Clinical trials involving EVs have most commonly been performed with native EVs prepared from culture supernatant of human cells, with 59% of these trials using mesenchymal stromal cells (MSCs) derived from bone marrow, adipose tissue or placenta (Heldring et al. 2015; Witwer and Théry 2019; Phinney and Pittenger 2017). Alternative cellular sources, such as neuronal stem and progenitor cells (Webb et al. 2018), blood and umbilical cord (Zhang et al. 2016), have been used with all studies involving the administration of a heterogeneous population of EVs. This is despite recognition that different EV subsets, as defined by phenotype, may exhibit distinctly different functional activities (Mizenko et al. 2021).

A major consideration for the EV therapeutic sector is not only ensuring the reproducible manufacture of products with consistent activity and safety profiles but also utilising fully characterised products, allowing for the correlation of outcomes with EV potency and attributes. This demand calls for the development of advanced technologies capable of analysing individual EV particles. With this new knowledge, it may be possible to tailor or fine tune EV manufacturing so that only active, beneficial vesicles are included in therapeutic formulations. Herein lies a significant opportunity for microfluidic‐based devices and methodologies, which can facilitate vesicle analytics and the selection and concentration of distinct EV subsets for the manufacture of tailored, bespoke products.

Another key consideration in EV product manufacture is the scale and cost of production. At present, vesicle‐laden supernatant collected from cell culture is most often used as a starting substrate for downstream processes that purify and concentrate EVs. Both adherent and non‐adherent cell types can be cultured using technologies that enable both scale‐up and scale‐out approaches to increase the volume of the starting substrate. However, most bioreactors and platforms were designed for the cell therapy sector or the production of cell‐secreted proteins, rather than for EVs. There is a need for innovative bioreactor design that provides control over culture conditions to maximise EV production, preserve EV integrity, retain biological cargo and ensure reproducible function and potency. Microfluidic systems, where cells are exposed to controlled shear stress, fluid flows and media supply have the potential to optimise EV production on a per cell basis. Key considerations for downstream processes that remove non‐vesicle contaminants and concentrate EVs include an interplay between preservation of vesicle integrity and function with process efficiency and vesicle recovery (Whitford and Guterstam 2019). The EV therapeutic sector has largely adopted technology used for the manufacture of non‐vesicle products such as proteins and small molecules for EV purification and concentration (https://www.bioprocessintl.com/separation‐purification/setting‐a‐cornerstone‐for‐platform‐purification‐of‐exosomes) as there are very few technologies specifically designed and optimised for large‐scale purification, concentration and formulation of vesicles.

Such technologies must ensure a robust control strategy for product consistency with a comprehensive toolkit of analytics for both in‐process control and final product characterisation. In today's regulated bioprocessing environment, such a control strategy is known as continuous process verification (CPV). Further details of CPV and the product development landscape for EV therapeutics are described in our previous publication entitled ‘Exosome Therapeutics: Academic curiosity or commercial reality’ (Exosome Therapeutics 2020).

**FIGURE 1:**
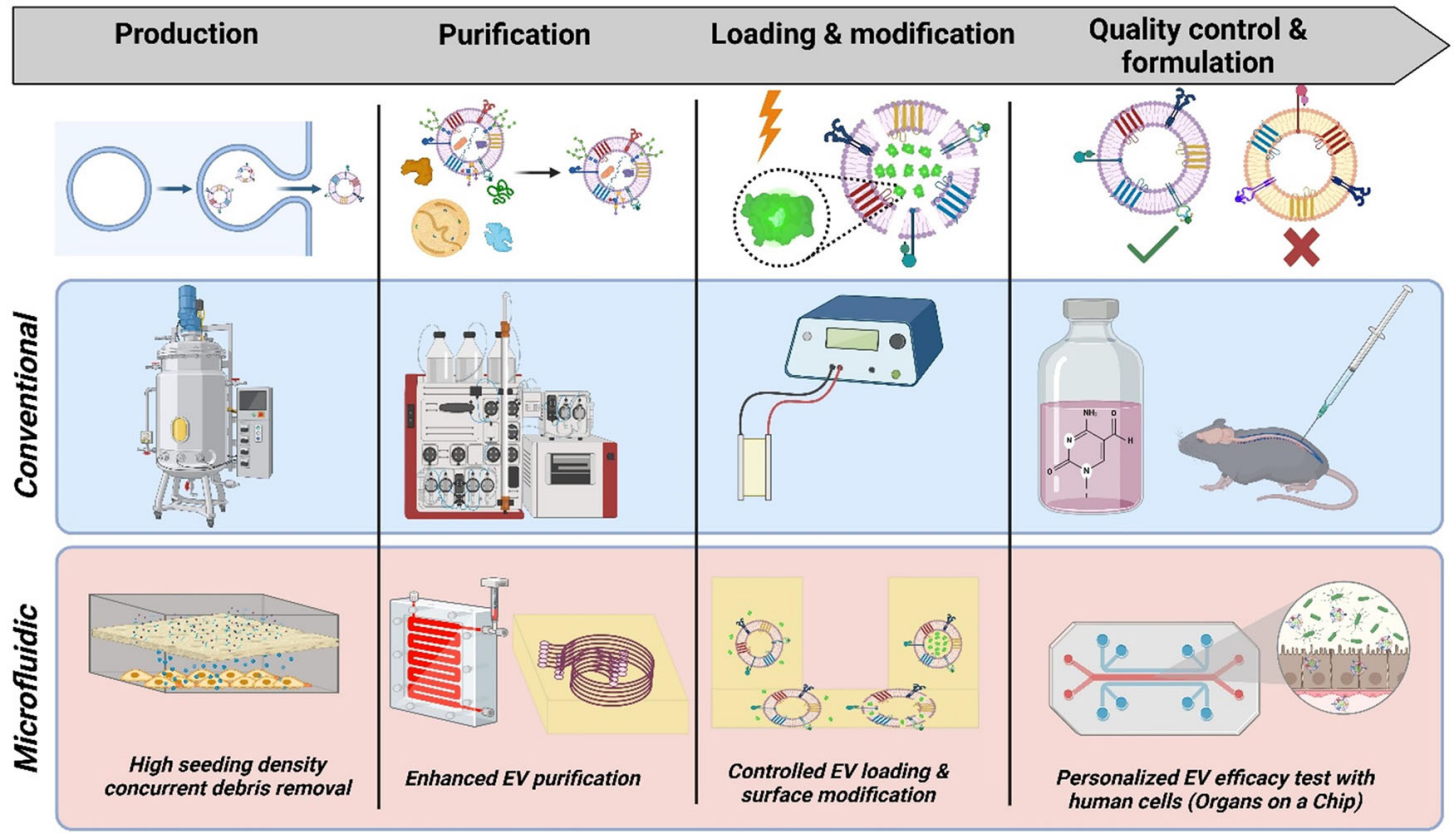
Schematic showing conventional extracellular vesicle (EV) manufacturing workflow (left to right) including EV production by cells grown in a bioreactor, followed by clarification, purification using tangential flow filtration (TFF) and size exclusion chromatography (SEC), loading and modification using systems such as electroporation (shown), followed by quality control and formulation. Microfluidics systems (left to right) can enhance the performance of this workflow at every step by increasing EV product purity, loading and surface modification and at the quality control step.

In this review, we highlight how microfluidic devices can be widely applied across the EV analytics and manufacturing process. We describe the advantages of this technology, progress to date and the opportunities and challenges afforded by using microfluidics as a platform for EV analysis, purification, concentration and product formulation. Although this review does not cover therapeutic mechanisms – extensively discussed elsewhere (Du et al. 2023; Wiklander et al. 2019; Kim et al. 2024) – our focus is on how microfluidic platforms can support the large‐scale, GMP‐compliant manufacturing of therapeutic‐grade EVs, thus addressing critical bottlenecks in clinical translation. In Figure 1, we describe a typical EV manufacturing pipeline involving harvesting of EV‐rich cell culture supernatant, followed by downstream purification and concentration steps such as tangential flow filtration (TFF) and size exclusion chromatography (SEC) and ending with product formulation. As discussed below, microfluidic technology can enhance performance at each stage of this pipeline. However, whilst microfluidic technology represents a flexible toolkit, its use for EV manufacture must be considered from a regulatory perspective particularly in terms of other therapies produced with microfluidic devices and how products using such systems will be evaluated by the FDA and other regulatory authorities worldwide.

## 2 Opportunities for Microfluidics in EV Manufacture

Microfluidics represents a transformative technology for EV manufacture, offering precise control over key steps, including production, purification, cargo loading, surface modification and quality control (QC). The multifunctionality, scalability and laminar flow characteristics inherent to microfluidic systems enable superior manipulation of EVs (Ngo et al. 2023), allowing efficient sorting, enrichment and functionalisation.

In EV production, compared to conventional bioreactors (Figure 1), microfluidic bioreactors provide controlled environments with adjustable shear stress and optimised nutrient delivery, enhancing yield and preserving EV integrity. For purification, microfluidic systems can facilitate size‐based, affinity‐based and hybrid isolation techniques, achieving high purity while minimising EV loss, as compared to conventional EV purification involving multistep processes of TFF and SEC, which have the potential for significant loss at each step. Cargo loading and surface modification are significantly improved through microfluidic platforms, which allow precise delivery of therapeutic agents into EVs and enable tailored functionalisation to enhance targeting efficiency. In comparison, bulk EV customisation methods may be less effective for heterogeneous populations of EVs, resulting in only a small subset of EVs being loaded (Rankin‐Turner et al. 2021). Lastly, the implementation of microfluidic‐based QC systems, allowing real‐time monitoring and analytics that ensure product consistency and compliance with regulatory standards, would revolutionise EV manufacture. This integration of microfluidics not only addresses current challenges in EV manufacturing but also paves the way for clinical translation and commercialisation of EV‐based therapeutics.

## 3 Microfluidic Devices for EV Manufacture

### 3.1 Microfluidic Bioreactors for Enhanced Production of EVs

With the rising interest in utilising EVs for therapeutic applications, there is an increasing demand for scalable, controlled and efficient EV production methods. Microfluidic bioreactors offer a promising solution by providing the ability to fine‐tune culture conditions, apply chemical and physical stimulation, and limit cell membrane disruption, ultimately enhancing EV yield and quality. This control is crucial for advancing EV therapeutics and meeting the standards required for clinical applications and is discussed in detail in this section.

#### 3.1.1 Design, Characterisation and Comparison of EV‐Producing Bioreactors

Efficient and scalable production of EVs requires well‐characterised cell culture systems, such as bioreactors, capable of supporting high cell viability, controlled culture environments and consistent EV quality. Various bioreactor platforms have been utilised for EV production, each with distinct design features and performance metrics. First, in terms of design mode, traditional platforms include T‐flasks, spinner flasks and hollow‐fibre bioreactors, which operate under either static or dynamic batch conditions. In contrast, microfluidic bioreactors typically employ continuous perfusion systems that enable precise fluid control and strict control over conditions, such as pH. Second, the culture format differs across systems. Although T‐flasks support 2D adherent cultures, microfluidic systems allow high‐density cultures within 3D structures, utilising confined channels or droplet‐based structures (Kronstadt et al. 2023). Third, key characterisation metrics include EV production per cell, cell viability and vesicle quality (e.g., size distribution, cargo retention). Conventional T‐flasks offer limited productivity, while hollow‐fibre and spinner flasks improve output but with greater risk of shear‐induced stress (Watson et al. 2016). Additionally, EV purity is often compromised in traditional systems due to bulk handling, cell debris and apoptotic vesicle release. Microfluidic systems can offer higher per‐cell productivity under low shear conditions, while preserving membrane integrity and cell viability. Microfluidic platforms can also offer reduced contamination risk via closed‐loop configurations and integrated filtration or separation modules. Table 1 provides an overview of microfluidic bioreactor platforms for EV production, detailing their cell sources, engineering strategies, production throughput and key quality metrics such as yield, marker expression, bioactive cargo and therapeutic application.

**TABLE 1:** Microfluidic bioreactor technologies for EV production.

Platform/study	Cell/sample type	Strategy/method	Throughput	Yield/efficiency	Markers/quality	Integrity/functionality	Bioactive EV content	Application	Ref
3D‐Printed Scaffold Perfusion Bioreactor	Human dermal microvascular endothelial cells (HDMECs)	3D‐printed scaffold + tubular perfusion bioreactor at 4 mL/min; ethanol conditioning	∼8.6 × 10¹⁰ EVs/24 h from 72 cm^2^ surface area	>100‐fold increase in EVs over flask; CD63+ EVs ∼14× increase; enhanced expression of MALAT1/HOTAIR	CD63, CD9, Alix, TSG101 (WB, ELISA); size by NTA (∼40–200 nm)	Low protein contamination; absence of calnexin, Ago2; retained bioactivity in gap closure assay	Proangiogenic lncRNAs (HOTAIR, MALAT1)	Vascularisation therapy, scalable therapeutic EV production	(Patel et al. 2019)
Herringbone Microfluidic Bioreactor + GelMA Microcarriers	BMSCs, NIH‐3T3, HepG2	GelMA porous microcarriers integrated into herringbone chip with 1 µL/min perfusion flow	Max ∼4.5 × 10⁷ EVs/mL (NTA) at 105 nm	∼21‐fold higher yield vs flask	DLS: 30–140 nm; TEM morphology; CD63, CD81 confirmed	Promoted wound closure, angiogenesis and reduced IL‐6 in diabetic rat model	Not specified (BMSC/FB origin suggested regenerative content)	Wound healing (diabetic rat model), scalable regenerative EV production	(Huang et al. 2023)
Flat‐Plate Bioreactor	hBM‐MSCs	Flow‐induced EV production via calcium signalling (1 mL/min perfusion); shear optimised	6.7× particle yield and 7× protein yield vs. static	CD9, CD63, CD81 (WB); no calnexin; TEM: spherical vesicles; NTA	Confirmed retention of MSC markers (CD105, CD44); no apoptosis; effective delivery to kidneys	Reduced apoptosis, ROS and injury markers (KIM‐1, NOX4); improved PCNA and tissue histology	Renal regenerative signals	Acute kidney injury (AKI) therapy (in vitro and in vivo)	(Kang et al. 2022)
3D‐Printed Scaffold Perfusion Bioreactor	Bone marrow‐derived MSCs and HEK293 cells	Bioreactor with pillared 3D‐printed scaffold; perfusion at 1–10 mL/min; optimal at 5 mL/min	Up to 83× increase over flask (NTA); ∼43× CD63+ EVs (ELISA); optimal at 5 mL/min	CD9, CD63, CD81, Alix, TSG101; no calnexin, Ago2, HSP90; NTA: ∼160 nm; TEM: intact morphology	Maintained in vitro angiogenic activity (gap closure, tube formation); improved in vivo healing vs. flask‐derived EVs	Enhanced CD31+ vessel formation in diabetic mouse wounds; increased gene expression of angiogenic factors	Not specified (MSC‐derived therapeutic cargo)	Wound healing (diabetic mouse model); scalable therapeutic EV manufacturing	(Kronstadt et al. 2023)
Microchannel‐Induced Nanovesicle Bioreactor	Murine embryonic stem cells (ES‐D3)	PDMS microchannels (200 µm × 5 µm) with 6.5 µL/min extrusion to induce vesicle shedding via shear stress	∼100 nm vesicles, ∼1/5th content of origin cells	RNA and protein content ∼20% of original cells; size control via channel length; optimal at 200 µm, 5 µm geometry	TEM: spherical bilayer vesicles; actin, Nanog, ICAM‐1 confirmed	Successfully delivered endogenous RNAs (Oct3/4, Nanog) into NIH‐3T3 cells; >80% uptake within 12 h (FACS, confocal)	Oct3/4, Nanog, actin mRNAs from stem cells	RNA delivery platform mimicking exosomes; nonviral gene transfer tool	(Jo et al. 2014)

Abbreviations: NTA, nanoparticle tracking analysis; TEM, transmission electron microscopy; WB, Western blot.

#### 3.1.2 Shear Stress in EV Producing Bioreactors

In microfluidic bioreactors, cells and EVs co‐exist, requiring careful adjustment of flow‐induced shear stress to maintain both cell and EV functionality. On the one hand, a safe shear stress level should be maintained to avoid damage, as excessive shear can disrupt cells and EVs’ membrane integrity and biological function. From another perspective, shear stress can act as a driving factor for increased EV production (Guo et al. 2021). Shear stresses up to 30 dynes/cm^2^ has been reported to be in the safe range for EV production without disrupting cell membranes (Thompson and Papoutsakis 2023). This limit might vary based on factors like cell/EV size, membrane composition and function. For instance, when exposed to a moderate shear stress range of 0.5–5 dyn/cm^2^ for 48 h, dental pulp‐derived MSCs demonstrated a 24‐fold increase in EV production, whereas adipose tissue‐derived MSCs showed only a two‐fold increase under the same conditions (Guo et al. 2021). To control shear within microfluidic bioreactors, a range of pumps can be applied. Syringe pumps are ideal for providing controlled shear rates for short durations whereas pressure‐controlled pumps are suitable for stable, pulsation‐free flow, presenting superior control over shear rates. Peristaltic pumps are also practical for long‐duration, continuous applications, although they introduce pulsation that could affect shear‐sensitive samples (Byun et al. 2014; Shemesh et al. 2015).

#### 3.1.3 Advantages of Microfluidic Bioreactors in EV Production

**FIGURE 2:**
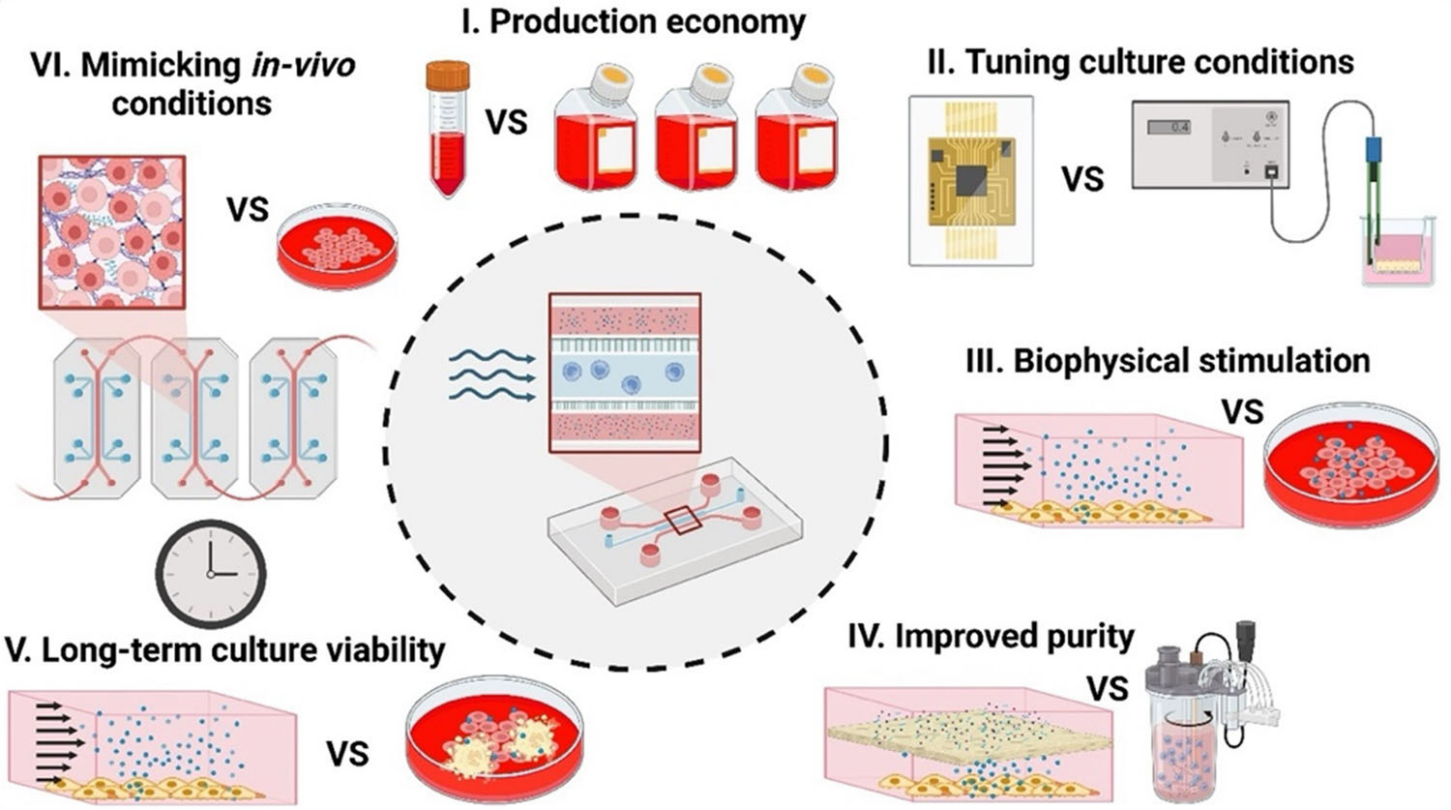
Advantages of microfluidic extracellular vesicle (EV) production over conventional culture include (A) production economy through minimising the use of culture medium and reagents; (B) tuning culture conditions using integrated sensors for real‐time monitoring of conditions such as temperature, dissolved oxygen and pH; (C) application of biophysical stimulation using controlled shear forces; (D) improved purity through embedded membrane filtration in the bioreactor; (F) long‐term culture viability through perfusion culture, removal of wastes and supplying fresh medium and (F) recapitulating the in‐vivo microphysiological conditions through 3D cultures.

In addition to the application of controlled shear stress, microfluidic bioreactors offer several other features and advantages compared to conventional EV production systems (Figure 2). These include enhanced production economy and cost effectiveness, tightly controlled culture conditions and cell stimulation, improved EV purity, increased cell viability and recapitulation of *in vivo* conditions. These are discussed in detail as follows.

I. Production economy

Microfluidic bioreactors offer a high surface‐to‐volume ratio, enabling the cultivation of large numbers of adherent cells in smaller volumes compared to traditional flask cultures and bioreactors. This helps to reduce the amount of medium and reagent consumption (Marques and Szita 2017; Pasirayi et al. 2011), while facilitating the production of concentrated EV products. These concentrated EVs can be processed more efficiently in microfluidic purification systems, leading to faster and potentially more cost‐effective production workflows.

II. Fine‐tuning culture conditions

Microfluidic bioreactor cultures can benefit from rapid control of oxygen tension, temperature and pH due to their smaller volumes (Rameez et al. 2014; Oomen et al. 2016). In addition, integration of oxygen, pH and temperature sensors (Wu et al. 2009; Azimzadeh et al. 2022; Giusti et al. 2017) allows fine‐control and real‐time monitoring, providing immediate feedback and adjustment. Microfluidic parallelisation can be used to test different microenvironmental conditions (e.g., varying oxygen concentration or pH) in a high‐throughput manner, which is more difficult to achieve in large‐scale conventional bioreactors.

III. Superior biophysical stimulation

Microfluidic platforms allow for highly controlled shear stress by adjusting flow rates, which can influence cellular behaviour, as well as the quantity, size and type of EVs produced (Kronstadt et al. 2023; Patel et al. 2019). This level of regulation is more difficult in conventional bioreactors, which typically offer less control over biophysical forces applied on cells. For example, Kang et al. (2022) showed that under low shear stress conditions (0.001 and 0.0001 dyn/cm^2^) in microfluidic bioreactor, total MSC EV protein increased significantly, rising 6.5‐fold after 24 h. This increase was accompanied by the upregulation of traditional MSC markers CD105 and CD44, alongside a concurrent downregulation of traditional EV markers CD81, CD63 and CD9. Shear stress also affects MSCs differentiation and viability. MSCs differentiated into osteoblasts under shear stress of 5–25 dyn/cm^2^ while their viability and proliferation were increased under shear stress of 10^−4^–10^−3^ dyn/cm^2^ (McAllister et al. 2000; Zhao et al. 2007).

IV. Reduced contamination and improved purity

In conventional bioreactors, the large volume and bulk nature of the system and manual multistep handling of reagents increase the risk of contamination. Limited control over culture conditions in conventional bioreactors may lead to increased cell death, an increase in apoptotic bodies and extracellular chromatin‐protein aggregates, which reduce the effectiveness of downstream EV purification and concentration (Wiest and Zubair 2020). In contrast, closed, microfluidic EV production platforms, where EVs are produced and stored continuously, significantly reduce the chance of contamination (Gwak et al. 2022). Microfluidic platforms can also use on‐chip filtration (Leclerc et al. 2004) or an aqueous two‐phase system (ATPS) strategy (Hardt and Hahn 2012) to remove proteins and other by‐products, further enhancing EV purity during production. The controlled flow conditions help to preserve the integrity and functionality of EVs, ensuring their therapeutic potential and preventing the release of contaminants into the culture medium.

V. Enhanced cellular viability and long‐term culture

Microfluidic systems allow for better control of cellular health over extended periods through controlling waste accumulation, nutrient delivery and gas exchange, thus increasing the overall yield of EVs. Through medium perfusion, cells in microfluidic systems can be maintained in long‐term culture, continuously producing EVs without the need for multiple culture cycles, enhancing the quantity of therapeutic EVs produced. Kronstadt et al. (2023) demonstrated that using a perfusion bioreactor culture system led to a remarkable 40–80‐fold increase in the production of MSC EVs compared to conventional static cell culture. Furthermore, MSC EVs produced via this advanced system were significantly more effective in enhancing wound healing in a diabetic mouse model, as evidenced by higher levels of CD31+ endothelial cells in wound bed tissue, compared to EVs derived from standard flask‐based cultures.

VI. 3D spheroid cultures using droplet microfluidics

Research has shown that 3D spheroid cultures enhance EV secretion in cells, which is attributed to hypoxia within the 3D spheroids, high cell density and non‐adherent cell morphology (Kim et al. 2018; Thippabhotla et al. 2019). Notably, the study revealed that as the size of the 3D spheroids increased, EV secretion decreased. This suggests that optimising spheroid size may be critical for efficient large‐scale EV production in 3D cultures. Droplet microfluidic devices have been able to produce mass numbers of uniformly sized spheroids, which can be employed for mass production of therapeutic EVs (Langer and Joensson 2020; Rima et al. 2022).

### 3.2 Scalable Isolation and Purification of EVs Using Microfluidic Systems

In any EV production pipeline, the presence of non‐EV particles – including apoptotic bodies (1–5 µm), microvesicles (100 nm–1 µm), protein aggregates (typically <20 nm), lipoproteins such as LDL (20–25 nm) and HDL (8–12 nm), and cell debris (Zhang et al. 2023) – poses a major challenge for downstream purification. These contaminants often overlap in size with sEVs (30–150 nm), making separation difficult. Microfluidic systems address this challenge through one or a combination of size‐based filtration, affinity capture and inertial or magnetophoretic forces, each tailored to remove a subset of impurities. For example, membrane‐based microfiltration targets large debris and apoptotic bodies, while ultrafine porous membranes and acoustofluidic technologies can exclude protein aggregates and lipoproteins. Immunoaffinity approaches further discriminate based on specific EV surface markers, helping eliminate non‐EV nanoparticles of similar size. The following sections describe how each of those microfluidic strategies is employed to perform these purification steps.

The terms microfluidic EV isolation and purification have been used interchangeably despite their different definitions. Purification techniques aim to separate EVs from various contaminants, including salts, biomolecules such as proteins and lipids, as well as larger apoptotic bodies. EV isolation is a broader term. Unlike purification methods, isolation techniques focus on reducing the complexity of the sample. Isolation might not only be used for purification purposes but also for in‐line analysis of EV subpopulations.

Microfluidic EV isolation has been extensively reviewed in the literature with most research focusing on diagnosis applications to detect EVs from small biological sample volumes. For clinical scale EV purification, however, samples have much larger volumes in the order of hundreds of millilitres to litres. This necessitates employing strategies to increase the microfluidic devices’ throughput. The performance metrics for any EV purification platform are purity, and recovery rate as defined as follows.

*Recovery rate* (Chen et al. 2020): The fraction of the total number of EVs recovered to the total number of EVs in the original sample solution.

*Purity* (Chen et al. 2020): The fraction of the isolated EVs among the collected particles of all sizes.

*Purity_protein_* (Brennan et al. 2020): The ratio of recovered EV particle counts to protein concentration.

*Purity_lipid particles_* (Brennan et al. 2020): Western blot analysis of lipoprotein markers APOB and APOE.

Current microfluidic EV isolation techniques show high sample purity, medium recovery rates but low input volume (Clos‐Sansalvador et al. 2022). The flow rate in these systems is in the order of tens of µL/min necessitating an extended period for large‐scale EV purification. The following section highlights advancements in EV purification that have been or can be implemented in a microfluidic platform to increase purification throughput and improve recovery. Table 2 summarises leading microfluidic platforms for EV isolation, comparing their input types, enrichment strategies, recovery rates, biomarker specificity, and downstream diagnostic or therapeutic applications.

**TABLE 2:** Microfluidic platforms for scalable EV isolation.

Platform/study	Cell/sample type	Strategy/method	Throughput	Yield/efficiency	EV markers used	Analysis method	Target molecule/cargo	Case specific application	Ref
ExoDisc	CCS (LNCaP), urine	Centrifugal microfluidic disc with 600/20 nm filters	∼1 mL processed in <30 min	>95% recovery, >100× mRNA vs. UC	CD9, CD81	ELISA	Urinary EV RNA	Clinical diagnostic from urine	(Woo et al. 2017)
EXODUS	Plasma, urine, CCS (HEK293T)	Dual‐membrane nanofiltration + harmonic oscillation	Up to 1–2 L/h (T‐2800 model)	Purity: >10⁸ particles/µg protein (https://www.atascientific.com.au/products/exodus‐t‐2800‐exosome‐isolation‐for‐gmp‐manufacturing/), >99% protein removal	CD63, CD81, TSG101, Alix, ApoA1	WB	mRNA, lncRNAs, ncRNAs	Liquid biopsy for urinary cancers	(Chen et al. 2021)
ExoTFF	Biofluids incl. CCS (UC‐MSCs)	TFF + cationic filtration in a syringe‐loop	11 mL/min including elution and recovery	933% increased purity; 124% increased concentration vs TFF	CD9, CD81, TSG101, Alix	Bioactivity, uptake	miR‐142 and let‐7a, Albumin↓	Clinical‐grade EV purification	(Kim et al. 2025)
ExoCPR	Plasma, CCS (HepG2)	Magnetic immunocapture+ Bubble mixing + immiscible phase separation (IFAST)	29 min total for 260 µL	75.8% capture, >90% purity	CD63	TEM, NTA, WB, uptake by 293T cells	GPC3+ EVs (from HCC patients)	Liquid biopsy for HCC; rapid high‐purity EV enrichment platform	(Guo et al. 2024)
ExoSD chip Immunomagnetic chip	CCS (MGC‐803), clinical serum samples	Magnetic separation with split‐stream flow	6 mL/h	>80% recovery, >83% purity	CD9, CD63	WB, NTA, TEM	EPCAM, CD97, HER2	Early non‐invasive gastric cancer detection	(Yu et al. 2021)
HiMEc ImmunoInertial Chip	Plasma lung cancer/CCS (A549)	Inertial sorting of bead‐bound EVs	≥1 mL/min	Recovery>88%, ∼94% purity (negative selection)	ApoA1/B100 (removal); CD63	TEM, NTA, Flow cytometry, Electrochemical signal	PD‐1 and PD‐L1 proteins on EV surface	EV‐based immunotherapy	(Kwon et al. 2025)
ExoDFF	Whole blood from healthy and T2DM subjects	Dean Flow Fractionation (label‐free)	Up to 80 µL/min (ExoDFFHT); ∼5 mL/h	3× UC yield	TSG101, flotillin and CD9, and ApoA‐1	WB, NTA, flow cytometry	CD41a+, CD45+ pro‐inflammatory EVs; total EV RNA (miRNA‐enriched)	Vascular inflammation in T2DM	(Tay et al. 2021)
Acoustic Nanofilter (SSAW)	CCS (OvCA429)	Surface acoustic wave‐based size‐fractionation	∼25 µL/min; continuous flow	>80% recovery for exosomes; >90% for larger MVs	CD63	WB, NTA	Flotillin‐1, HSP70, HSP90	Exosome/MV isolation from cell culture and stored blood	(Lee et al. 2015)
ExoSponge	CCS (H3255), plasma from NSCLC patients	Porous PDMS sponge; Annexin V and anti‐CD63 affinity capture	∼1.4–1.9 mL per device; ∼5–10 mL processed in 40 min	9.4 × 10⁹ EVs/mL; 2.9× higher yield vs. UC; purity up to 90.9%	CD63	LC‐MS/MS metabolomics validated, NTA, TEM, WB	Phenyacetylglutamine (PAG), choline, betaine, MMA (exosomal metabolites)	Liquid biopsy for NSCLC	(Marvar et al. 2023)
OncoBean Chip	CCS (Patu8988t), Plasma (NSCLC)	Radial flow + bean‐shaped microposts (anti‐CD9/63/81)	Up to 10 mL/h (cell media); 1.2 mL/h (plasma)	High recovery; miRNA and protein confirmation	CD9, CD63, CD81	Flow cytometry (CD63, CD81); SEM, NTA; Uptake	miR‐21, miR‐155, miR‐200	Pancreatic cancer EV miRNA profiling	(Lo et al. 2020)

Abbreviations: CCS, cell culture supernatant; MV, microvesicle; NSCLS, nonsmall cell lung cancer; PDMS, polydimethylsiloxane; T2DM, Type 2 diabetes mellitus; TFF, tangential flow filtration; UC, ultracentrifugation.

#### 3.2.1 Microfiltration

Microfluidic devices (as listed below) that incorporate nanoporous interfaces allow selective isolation of EVs based on their size. These interfaces can be designed to exclude larger particles, such as apoptotic bodies and cell debris and smaller particles such as proteins and free nucleic acids while retaining EVs.

- Exodus (Exodus Bio, USA)

**FIGURE 3:**
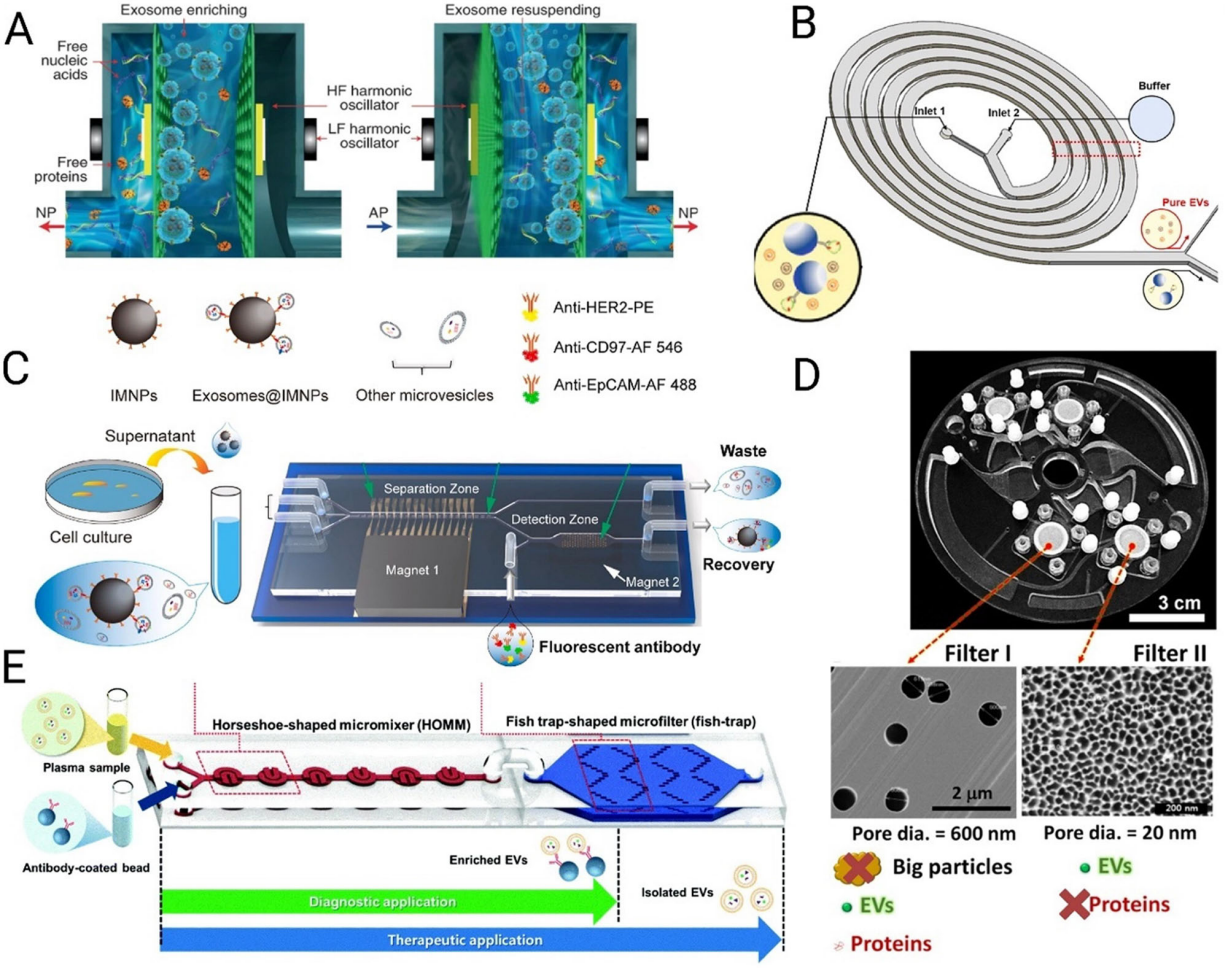
Scalable microfluidic extracellular vesicle (EV) purification (A) Exodus, reproduced from (Chen et al. 2021), with permission from Springer Nature; (B) Negative inertial, reproduced from (Kwon et al. 2025), with permission from Elsevier; (C) Immuno‐magnetic isolation, reproduced from (Yu et al. 2021), with permission from Elsevier; (D) Exo‐disc, reproduced from (Woo et al. 2017), with permission from the American Chemical Society and (E) Immuno‐trapping purification, reproduced from (Gwak et al. 2022), with permission from the Royal Society of Chemistry.

A major drawback of a membrane EV filtration technique is pore saturation. Exodus resolves this issue using a dual‐membrane nanofiltration system that integrates periodic negative pressure oscillation (NPO) and double‐coupled ultrasonic harmonic oscillations (HOs) and effectively removes free proteins and nucleic acids (Figure 3A) (Chen et al. 2021). Exodus system has been adopted by several leading medical centres in the United States for isolation of EVs for diagnostic and therapeutic applications. They claim their T‐2800 system can isolate EVs from 10 L MSC culture medium at 1–2 L/h with purity of >10^8^ particle/µg protein, protein removal efficiency of >99% and EV concentration of >1 × 10^11^ particles/mL (https://www.atascientific.com.au/products/exodus‐t‐2800‐exosome‐isolation‐for‐gmp‐manufacturing/).

- Formulatrix µPulse System (Formulatrix, United Arab Emirates)

The µPulse TFF system is another microfiltration product for fully automated EVs concentration (https://formulatrix.com/pulse‐tangential‐flow‐filtration/). The system's electronically controlled transmembrane pressure (TMP) and backpressure valves ensure precise pressure regulation, mitigating the risk of sample damage and membrane fouling. Its high‐precision weight sensors enable accurate volume tracking with ±0.2 mL accuracy, preventing over‐concentration and ensuring buffer exchange efficiency.

The µPulse system is designed for scalability, accommodating sample volumes ranging from 1 to 100 mL and supporting buffer exchange of up to 250 mL. Its throughput is up to four times faster than traditional dead‐end filtration, with a permeate flow rate significantly higher due to a filtration membrane that is 50%–75% larger than conventional systems. The system's low hold‐up volume of 0.65 mL minimises sample loss, achieving close to 100% sample recovery.

The µPulse system uses disposable chips that are available in a range of molecular weight cutoffs (MWCOs) from 5 to 300 kDa, allowing flexibility across different EV workflows. The system has been demonstrated to efficiently process EVs with minimal contamination while preserving their integrity, making it a robust alternative to traditional filtration techniques.

#### 3.2.2 TFF‐Inspired Systems With Microfluidic Integration Potential

Microfluidic tangential flow filtration (µTFF) systems continuously filter EVs while maintaining a steady flow of liquid sample (Han et al. 2021). However, despite their precision and scalability potential, current µTFF platforms face major limitations in processing throughput. Employing TFF in large‐scale applications typically requires increasing membrane surface area and optimising flow dynamics to accommodate higher sample volumes. In this context, two recent modular systems, ExoTFF and the Daicel Exosome Purification System, offer instructive models for overcoming such challenges.

- ExoTFF (Microgentas, South Korea; HannsaBioMed Life Sciences, Estonia)

ExoTFF is a hybrid EV isolation platform that combines electrokinetic filtration (ExoFilter) with size‐based TFF to improve both yield and purity (Kim et al. 2025). The ExoFilter consists of a multilayered, positively charged mesh embedded in a syringe, which selectively captures negatively charged EVs without relying on pore size exclusion. This unit is coupled to a TFF module containing hollow fibres with a 50 ± 10 nm pore size (800 kDa MWCO). By alternating sample flow between the two modules in a reciprocating, closed‐loop process, ExoTFF exploits charge‐ and size‐based selectivity to remove proteins and lipoproteins, including HDL, with high efficiency. Compared to TFF alone, ExoTFF achieved a 933% increase in purity ratio and a 124% increase in particle concentration, highlighting a strong synergistic effect between the two filtration mechanisms. The system also demonstrated >80% EV recovery within 10 min for 10 mL samples and processed 500 mL samples in 45 min at 11 mL/min. EVs retained functional bioactivity and exhibited strong marker expression (CD9, CD81, Alix, TSG101), minimal albumin contamination and efficient uptake by target cells, confirming their integrity postisolation.

- Exosome Purification System (Daicel, Japan)

This system offers a novel solution for scalable EV isolation and purification by utilising a combination of adsorbent treatment and membrane‐based filtration methods. It employs a positively charged porous granular adsorbent that selectively binds negatively charged impurities, such as proteins and nucleic acids, while preserving the integrity of EVs. The adsorbent material's optimised pore size and functionalisation allow for high specificity in removing unwanted contaminants from culture supernatants. Following the adsorbent treatment, the system integrates hollow‐fibre TFF for further concentration and purification. The hollow‐fibre membranes have molecular weight cutoffs ranging from 100 to 1000 kDa and feature a membrane surface velocity of 0.3–2 m/s. This dual‐stage process enhances EV recovery and ensures high purity, with reported concentration ratios exceeding five‐fold and recovery rates greater than 50% (Nakatsuka et al. 2024). The Daicel system also enables precise process control, supporting alternating flow directions in TFF to improve separation efficiency and prevent membrane fouling. The modular design permits scalability, making it suitable for both laboratory‐scale and industrial‐scale applications in EV manufacturing.

#### 3.2.3 Inertial and Immuno‐Inertial Systems

Inertial microfluidics is efficient in the isolation of microparticles and cells (Carlo 2009; Kuntaegowdanahalli et al. 2009). However, applying inertial microfluidics for EV purification at high throughput is limited due to the EVs nanometre size range and their size overlap with lipoproteins and nucleic acids present in biological samples. To address this issue, EVs can be captured on the surface of functionalised microparticles in a membrane‐free inertial isolation system. This allows sample processing at flow rates as high as 500 mL/min by scaling out parallel inertial microfluidics devices (Warkiani et al. 2015) and is likely to be applicable for EV isolation. Two approaches can be applied: firstly, in positive selection, microparticles functionalised with antibodies against EV proteins such as tetraspanins, CD9, CD63 or CD81 can be used for immune‐inertial isolation. A drawback of this method is that the recovery rate depends on the expression of these canonical EV proteins. However, an advantage of this method is the isolation of EVs with specific biomarker expression. For instance, Razavi Bazaz et al. (2023) conjugated anti‐PD‐L1, anti‐CD‐EpCAM and anti‐EGFR to 3, 10 and 20 µm beads and fractionated these beads at the outlet due to their differential migration in a zigzag microfluidic device, resulting in 90% capture efficiency. A high flow rate of 1 mL/min can process up to 1 L of EV sample in ~16 h. Secondly, EVs can be purified in an immune‐inertial device through a negative enrichment scheme. For instance, lipoproteins HDL and LDL were removed from the EV sample through microparticles functionalised with Apo‐A1 and Apo‐B100 antibodies with 94% purity (Kwon et al. 2025) (Figure 3B). The presented microfluidic device uses 10:1 sheath to sample flow ratio with sample flow rate of 50 µL/min. However, it is possible to upscale this platform with more parallel units to increase the throughput to the minimum of 1 mL/min (1 L/16 h). A purely inertial system, Exosome Dean Flow Fractionation (ExoDFF), has been reported as a label‐free method for sEV purification, removing the need for immunocapture and thereby enabling the isolation of all sEVs regardless of specific marker expression (Tay et al. 2021). ExoDFF achieves a throughput of approximately 20 µL/min per chip, with four parallelised subunits reaching a combined throughput of 80 µL/min, allowing for the processing of 5 mL of whole blood in an hour. This scalability makes it suitable for large‐scale sEV purification. The system yields sEV recovery rates three times higher than ultracentrifugation (UC) and achieves high purity by effectively separating nanoscale EVs (50‐200 nm) and medium‐sized EVs (200 nm‐1 µm). Moreover, its gentle, shear‐free sorting mechanism preserves the functional integrity of sEVs, making it ideal for downstream applications.

#### 3.2.4 Immunomagnetic and Acoustic Systems

Magnetic fields have been incorporated in microfluidic cell isolation techniques where capture beads bind to target cells and magnetic force isolates bead‐bound cells by deviating them along the channel length in a phenomenon known as magnetophoresis (Forbes and Forry 2012). Since the effect of magnetic forces is solely dependent on the presence of magnetic particles, this separation method can be equally applied to nanoparticles with overlapping sizes, such as EVs and lipoproteins, provided the magnetic bead targets EVs specifically. Incorporating magnetic field at the microscale ensures application of high magnetic field gradient across the microchannel that eventually translates to high efficiency and high purity recovery. Like other microfluidic approaches, parallelisation of multiple chips will be required to increase the throughput to liters of input sample. However, magnetic field strength can be increased to ensure EVs can be purified even at high flow rates. Yu et al. (Yu et al. 2021) presented an immunomagnetic microfluidic platform with high sEV recovery (>80%) and purity (>83%) at the injection rate of up to 6 mL/h for a single chip (Figure 3C). A key to their success in achieving high purity and efficiency is the introduction of a microfilter that physically divides the fluid into three streams, ensuring all EVs experience the same forces as they move along the magnetic field. A 10X parallelisation of this flow rate gives a flow rate of 60 mL/h allowing purification of 1 L of EV samples in ~16 h.

Acoustofluidic systems have emerged as effective tools for EV isolation and purification, leveraging standing surface acoustic waves (SSAWs) or bulk acoustic waves (BAWs) for label‐free particle manipulation (Lee et al. 2015). However, their operational flow rate remains low. For instance, Wu et al. (2017) developed an acoustofluidic device that isolates EVs with a recovery rate of 82.4% and purity of 98.4% for particles under 140 nm; however, the operating flow rate is only 4 µL/min. Hao et al. (2020) introduced silica nanoparticle‐assisted acoustic microfluidics, enhancing isolation efficiency by amplifying the acoustic forces acting on EV‐nanoparticle complexes. Although throughput for acoustofluidic system remains lower than that of magnetic or inertial methods, the purity and efficiency improvements highlight its potential for specific applications in EV manufacturing.

#### 3.2.5 Exo‐Disc

Exo‐disc is a microfluidic centrifugal system coupled with membranes (Figure 3D). Samples move across multiple membrane‐embedded chambers on a disc (Woo et al. 2017). As the disc rotates, the mild centrifugal force (500 × *g*) drags the sample radially outwards. The first set of membranes removes large cell debris and particles, and the second set filters out the proteins and smaller particles. There are two aspects of this platform that make it a potential candidate for large scale EV purification. First is high purity: the architecture of the system allows on‐demand liquid routing, a capability that allowed washing the residual proteins with as many washing cycles as needed leading to high sample purity of 6 × 10^8^ particles/µg proteins. Second, the high parallelisation capability: The Exo‐disc system also shows >95% recovery of EVs from cell culture media at flow rate of 33 µL/min. However, high throughput purification can simply be achieved by adding more filtration units on a disk and stacking multiple disks in a compact purification package. Whilst promising, this technology may result in co‐purification of non‐EV particles that are similar in size to EVs. Therefore, subsequent EV purification methods would be required.

#### 3.2.6 Immuno‐Trapping via Microbeads

Functionalised beads that capture EVs can also be physically trapped. This allows washing off any unbound nanoparticles and molecules. It also enables the processing of more samples and larger input volumes given that physical traps can capture beads even at high flow rates. One modular enrichment and harvest microfluidic platform (Gwak et al. 2022) uses a microfluidic horseshoe‐shaped orifice micromixer (*HOMM*) to selectively capture EVs on target‐specific antibody‐coated beads (Figure 3E). The beads were then captured by a fish‐trap‐shaped microfilter unit to elute and purify the affinity‐selected EVs. Captured EVs were released by the elution buffer [0.1 M glycine–HCl (pH 3)] and neutralised at pH 7.4 by using 1 M Tris–HCl (pH 9.0). An EV capture efficiency of >97% was reported for samples with EV concentrations of 10^9^–10^11^. The flow rates of up to 200 µL/min were reported, which can be easily upscaled to 1 mL/min with a 5‐parallel chip configuration and to 5 mL/min with a 25‐parallel chip configuration, enabling processing of 1 L of EV sample within 3 h.

#### 3.2.7 High Throughput Immunocapture Systems

Recent advances in microfluidic immunocapture platforms have focused on improving scalability, yield and purity of EV isolation to meet clinical and manufacturing demands. The *ExoSponge* device, a porous 3D PDMS scaffold functionalised with anti‐CD63 or Annexin V, enabled rapid, high‐yield EV capture from up to 10 mL of plasma or culture medium without the need for cleanroom fabrication (Marvar et al. 2023). Compared to UC, ExoSponge achieved a >210% increase in EV yield, and significantly enhanced purity (up to 90.9% vs. 76.5% with UC). The device isolated 9.40 × 10⁹ EVs/mL from lung cancer plasma and retained functional integrity of the EVs for downstream LC‐MS/MS‐based metabolic profiling.

Another scalable immunocapture platform is the *OncoBean chip*, which uses a radial flow microfluidic design and bean‐shaped microposts functionalised with desthiobiotin‐conjugated antibodies against CD9, CD63 and CD81 (Lo et al. 2020). This system demonstrated high capture efficiency from both plasma and culture medium (up to 10 mL processed in 1 h) with recovery of functional EVs confirmed via protein (CD9 and β‐actin) and miRNA (miR‐21, miR‐155 and miR‐200) analyses. Moreover, it enabled the release of intact EVs through biotin‐displacement of desthiobiotin, supporting downstream functional assays including cell uptake and flow cytometry profiling.

#### 3.2.8 Combining Size and Affinity‐Based Methods for EV Isolation and Purification

Combining size‐based and affinity‐based techniques in microfluidic systems is emerging as a powerful approach for improving EV isolation and purification. Size‐based methods like deterministic lateral displacement (DLD) effectively sort EVs in the 50–150 nm range, while affinity‐based methods using antibodies, such as anti‐CD63 or anti‐CD81, specifically target EVs expressing specific markers (Liu et al. 2021; Casadei et al. 2021). When integrated, these approaches significantly enhance purity, achieving levels over 90% particle‐to‐protein ratios, as demonstrated by systems like the Immuno‐Inertial microfluidics platform (Razavi Bazaz et al. 2023). The ExoCPR system combines immunomagnetic bead capture with immiscible filtration to yield EVs with over 90% purity in under 30 min, significantly reducing sample handling and contamination (Guo et al. 2024). Similarly, crossflow microfiltration devices incorporating size‐selective membranes with antibody‐functionalised channels achieved 76% recovery of EVs from conditioned media and improved isolation throughput (Casadei et al. 2021). This integration also minimises nonspecific binding and contamination, critical for ensuring therapeutic EV consistency and functionality.

### 3.3 On‐Chip EV Loading and Modification

**FIGURE 4:**
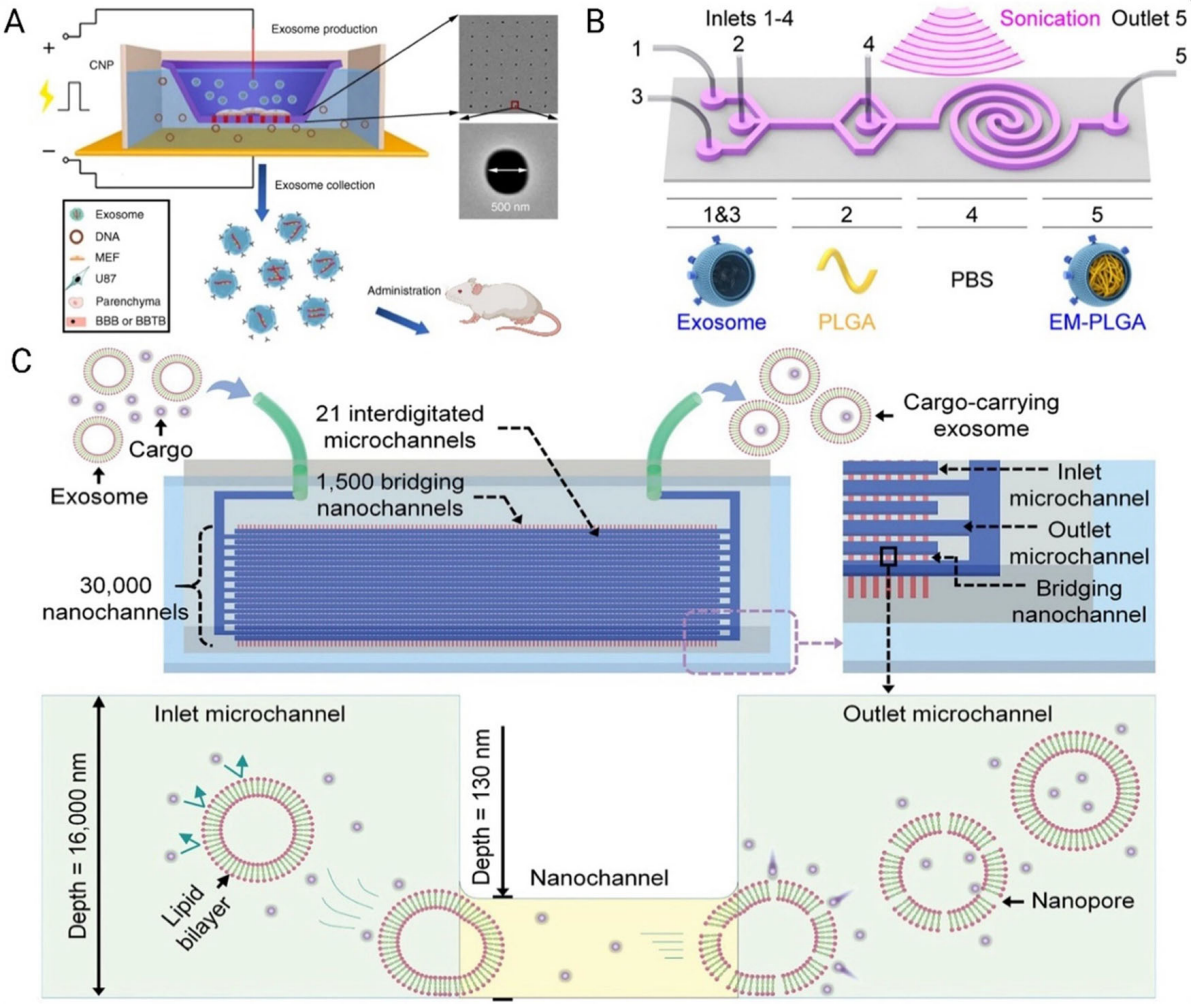
Microfluidic extracellular vesicle (EV) loading using (A) cellular nanoporation (CNP) biochip, reproduced from (Yang et al. 2020), with permission from Springer Nature; (B) microfluidic sonication, reproduced from (Liu et al. 2019), with permission from the American Chemical Society and (C) shear‐induced sEV nanoporation (ENP) device, reproduced from (Hao et al. 2021), with permission from John Wiley and Sons.

Microfluidic platforms provide efficient and precise methods for loading therapeutic cargo into EVs and modifying their surface properties, enhancing their functionality for targeted therapeutic applications (Figure 4). These on‐chip techniques enable controlled cargo loading, increased loading efficiency and uniform surface modifications, while preserving EV integrity. Table 3 also summarises recent microfluidic strategies for EV loading and surface modification, highlighting platform designs, cargo types, loading efficiencies and functional performance in therapeutic models.

**TABLE 3:** Microfluidic EV loading and modification strategies.

Platform/study	Cell/sample type	Strategy/method	Target molecule/cargo	Throughput	Yield/efficiency	Markers/quality	Integrity/functionality	Application	Ref
Microfluidic Electroporation (Exo‐Load)	SF7761 stem cells‐like‐ and U251‐GMs	Saponin permeabilisation in a sigmoid channel	Doxorubicin (DOX), Paclitaxel (PTX)	50 µL/min (linear); ∼1.2 × 10¹⁰ exosomes/h	DOX loading: 19.7% (SF7761); up to 31.98% (U251, sigmoid Exo‐Load); PTX: 17.7%	CD63	Preserved structure; improved uptake in glioma cells; (immunogold EM); NTA, TEM	Targeted delivery of anticancer drugs to glioma	(Thakur et al. 2020)
Microfluidic Sonication for Exo‐NP Fusion	A549 lung cancer exosome	Microfluidic sonication in an ultrasonic bath	Exosome membrane proteins (CD63, CD47); DiO dye cargo	174 mL/h	∼90.5% membrane‐coating efficiency with sonication (vs. 47.3% without)	CD9, CD63	Maintained membrane integrity; fusion confirmed CD47 (dot blot); DLS, TEM, zeta potential analysis	Tumour‐specific drug delivery via immune evasion; homotypic targeting using coated NPs	(Liu et al. 2019)
ENP (Exosome Nanoporator)	A549 (nonsmall cell lung cancer)	Nanofluidic chip with 30,000 nanochannels; shear‐induced membrane poration	Dextran (3, 10 kDa); DOX·HCl	8 µL/min; ∼4.3 × 10⁸ exosomes/h (8 µL/min; 9.0 × 10⁸ exo/mL)	∼37% (3 kDa dextran); 94.4% recovery; 4.9 µg DOX loaded into 1.7 × 10⁸ exosomes	PKH26 lipophilic dye	Maintained morphology; enhanced uptake; DOX delivery induced 54% cell death and tumour spheroid shrinkage	Tumour penetration	(Hao et al. 2021)
Click Chemistry Microfluidic GUVs	Synthetic phospholipids (POPC/DOPC)	SPAAC (strain‐promoted alkyne–azide click chemistry)	DSPE‐DBCO (a lipid tagged with a strained alkyne group)	∼80 µL/min (outer aqueous)	Not specified	Fluorescence imaging for conjugation	Bending rigidity measurement confirmed vesicle asymmetry	Membrane engineering	(Karamdad et al. 2016)
Photocleavable Antigen Decoration	Human leukocytes; JAWSII dendritic cells	On‐chip conjugation via photocleavable linker and magnetic beads	gp‐100, MART‐1, MAGE‐A3 tumour antigenic peptides	∼1.2 × 10⁹ exosomes/h (from 4 × 10⁴ cells)	∼95% antigen‐conjugated EV release	MHC‐I+ exosome binding; fluorescence tracking	Enhanced THP‐1 uptake; 2× IFN‐γ secretion; 30% CD8⁺ T cell proliferation in gp‐100‐loaded group	Immunogenic exosome engineering personalised cancer immunotherapy	(Zhao et al. 2019)
Lipid Insertion in Microfluidics	Milk‐derived exosomes	Rapid PEG and HFQ lipid insertion via microfluidic mixing	(EK)4‐KK and (SG)5‐RGD HFQ lipids	1 mL/min; <2 min mixing time	Improved targeting vs. bulk mixed EVs; size stability	(CD81); Zetasizer for size and polydispersity index	Preserved morphology; enhanced lysosomal uptake	Engineering EVs with stealth and targeting capability	(Geng et al. 2024)
µDES (Microfluidic Droplet‐based Electroporation System)	HEI‐OC1 cells; MSCs; Shaker‐1 mice fibroblasts	Droplet‐based low‐voltage continuous‐flow electroporation	CRISPR gRNA:Cas9 RNP targeting Myo7a mutation	∼30 mL/h per device	∼80% Cas9 loading efficiency; >10× efficiency and >1000× throughput vs. cuvette electroporation	CD81, TSG101, Alix (WB, AuNP‐TEM); NTA, zeta potential	>50% editing of Myo7ash1 allele in vitro; significant hearing restoration in vivo	Allele‐specific gene editing for dominant progressive hearing loss in vivo	(Pan et al. 2023)
Cellular Nanoporation (CNP)	MEFs, BMDCs, U87 glioma, GL261 cells	Nanochannel electroporation of cells to transcribe and secrete mRNA‐loaded exosomes	PTEN mRNA; glioma‐targeting peptides on CD47	∼10¹^2^ exosomes per 3‐day cycle per 10⁶ cells	>50× EV yield vs BEP; >10^3^× mRNA loading efficiency	WB (CD9, CD63, TSG101); Cryo‐EM; DLS; qRT‐PCR	Intact mRNA; restored PTEN expression; high tumour uptake; low immunogenicity	Systemic mRNA delivery across blood–brain barrier for glioma therapy	(Yang et al. 2020)

#### 3.3.1 Methods for Loading Therapeutic Cargo Into EVs

- Electroporation

Microfluidic electroporation chips apply a precisely controlled electric field to temporarily permeabilise the cell membrane, allowing therapeutic agents such as small RNAs, drugs or peptides to enter cells and the subsequent EVs produced from those cells. Microfluidic electroporation systems can contribute to enhancing loading efficiency (Yang et al. 2020) and maintain EV integrity and transfection consistency (Pan et al. 2023). In research by Yang et al. (2020), DNA plasmids were delivered into cells through nanochannels using transient electrical pulses and enabled the release of EVs loaded with functional mRNAs (Figure 4A). This microfluidic cellular nanoporation method increased EV production 50‐fold and improved mRNA loading efficiency by over 1000‐fold, achieving 2–10 intact mRNA molecules per EV. Microfluidic localised control of the electric field intensity and pulse duration ensures consistent cargo loading and minimises unwanted side effects, such as RNA degradation or leakage of luminal contents.

- Sonication

Sonication‐based EV loading uses ultrasound waves to temporarily destabilise the lipid bilayer of EVs, facilitating the encapsulation of hydrophobic drugs and larger molecules. In bulk sonication, the unpredictable quantity and distribution of microbubbles, combined with the variability in the shear stress produced, increases the vulnerability of cells to damage (Liu et al. 2012). Microfluidic sonoporation resolves this, providing fine control over ultrasound intensity and exposure time. Liu et al. (2019) used ultrasonic waves to disrupt and reassemble EV membranes around drug‐loaded nanoparticles, forming a core–shell structure (Figure 4B). Acoustic forces (up to 200 kPa) enabled delicate EV membrane rupture and fusion. This method achieved a 93% membrane coating efficiency, much higher than the typical <30% efficiency of conventional EV drug‐loading methods.

- Passive Loading

Some therapeutic molecules, such as small RNAs, lipophilic drugs, or proteins, can passively diffuse across the EV membrane when incubated under optimised conditions. Microfluidic incubation chambers allow for precise control of incubation time, temperature, shear force and concentration gradients, which may increase the loading yield, compared to conventional passive loading methods. For example, Hao et al. (2021) describe an sEV nanoporator (ENP), a device that enables high‐throughput cargo loading into sEVs by guiding them through nanochannels that temporarily disrupt their membranes via mechanical forces and fluid shear (Figure 4C). This process allows cargo uptake while preserving sEV integrity. With 30,000 nanochannels, the ENP efficiently processes samples, and the loaded sEVs effectively deliver drugs to nonsmall cell lung cancer cells, inducing cell death.

- Saponin‐Based

Saponin‐based sEV drug loading in microfluidic devices demonstrates distinct advantages over traditional bulk methods by reducing the risk of vesicle aggregation or degradation associated with excessive saponin exposure. Exo‐Load is a microfluidic device that enables precise control of shear stress and membrane permeabilisation during drug loading (Thakur et al. 2020). By employing optimised parameters, such as a flow rate of 50 µL/min, the Exo‐Load achieved a doxorubicin loading efficiency of 19.7% for SF7761 glioma‐derived sEVs, outperforming conventional techniques. Furthermore, the incorporation of a sigmoid channel design enhanced the loading efficiency to 31.98% for U251 glioma‐derived sEVs at a reduced flow rate of 12.5 µL/min, highlighting the potential of microfluidic systems to improve drug encapsulation in sEVs through tailored design and operational parameters.

#### 3.3.2 Methods for Surface Modification of EVs

- Lipid Insertion

**FIGURE 5:**
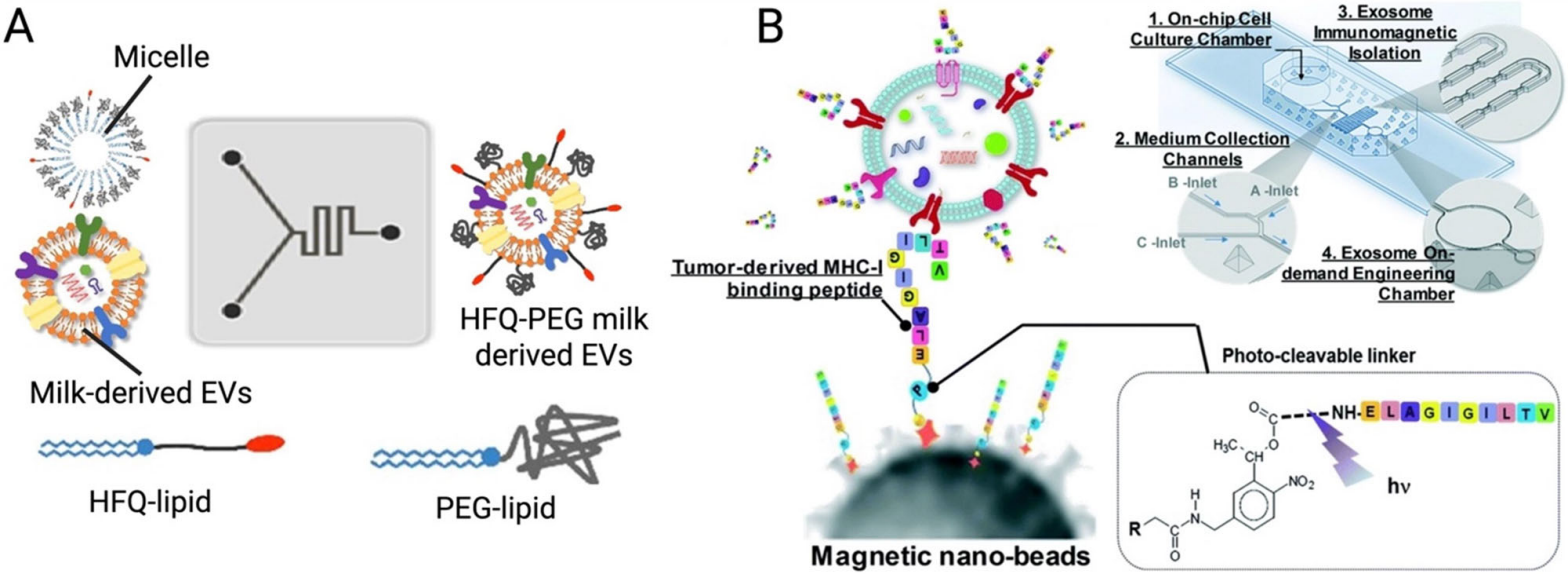
Microfluidic surface modification of extracellular vesicle (EV) through (A) On‐chip lipid insertion, reproduced from (Geng et al. 2024) with permission from Elsevier; (B) On‐chip binding to a magnetic capture bead using a photo‐cleavable linker, reproduced from (Zhao et al. 2019) with permission from Royal Society of Chemistry.

Microfluidic lipid insertion for EV surface modification offers significant advantages over bulk methods by ensuring rapid and uniform incorporation of lipophilic molecules, minimising vesicle destabilisation or aggregation. Microfluidic mixing can simultaneously incorporate polyethylene glycol (PEG) and KK‐ or RGD‐modified high‐functionality lipids (HFQs) into purified milk‐derived EVs (Geng et al. 2024) (Figure 5A). This approach generated modified EVs ranging from 160 to 165 nm and with a polydispersity index (PDI) of 0.26, which demonstrated superior cellular targeting compared to EVs generated by bulk methods. This method allowed precise control over the lipid ratio and the 5KK‐PEG EVs were significantly better at binding to PC9 and A549 cells. The short mixing time (<2 min) also reduced the risk of degradation or drug leakage, demonstrating microfluidics as a scalable, efficient method for EV functionalisation.

- Click Chemistry‐Based Conjugation

Microfluidic systems enable precise and efficient surface modification of EVs using click chemistry, offering advantages over bulk methods in speed, selectivity, control of reaction conditions and postconjugation purification. In a study, Karamdad et al. (2016) utilised a microfluidic platform to generate asymmetric giant unilamellar vesicles (GUVs) and demonstrated successful modification through strain‐promoted alkyne‐azide click chemistry (SPAAC). The microfluidic approach allowed selective incorporation of azide or alkyne‐functionalised lipids on specific leaflets of the vesicle membrane, achieving high spatial resolution. Fluorescence analysis confirmed the conjugation efficiency, and the reaction was completed within 25 min. This precise and controlled environment minimised vesicle destabilisation and allowed scalable, reproducible production.

- Antigenic Peptide Decoration via Photo‐Cleavable Microfluidic Conjugation

A microfluidic cell culture chip enabled real‐time surface modification of EVs with tumour‐specific MHC‐I‐binding peptides for immunotherapy applications (Zhao et al. 2019) (Figure 5B). In this system, leukocytes were cultured on‐chip, and secreted EVs were continuously harvested, captured via magnetic beads functionalised with a photo‐cleavable linker, and conjugated with melanoma antigens such as gp‐100, MART‐1 and MAGE‐A3. Upon UV exposure, intact surface‐engineered EVs were released with high efficiency (∼95%) and retained their ability to stimulate antigen‐specific CD8+ T cell proliferation. This workflow integrated cell culture, antigenic engineering and recovery in a closed‐loop microfluidic environment, offering a scalable and precise method to generate immunogenic exosomes for cancer therapy.

## 4 Implementing Microfluidics in Quality Control and Characterisation of EVs

### 4.1 Importance of Quality Control (QC) in GMP Manufacturing of EVs

In EV‐based therapeutics, QC is essential for consistent, effective and safe production, and meeting regulatory standards for clinical use. Good manufacturing practice (GMP)‐accredited facilities are required to produce EVs with reproducible therapeutic properties at every manufacturing step (Figure 1), and this demands rigorous, standardised QC protocols (Théry et al. 2018). Characterisation of EVs‐including size, protein markers, molecular cargo and functional properties, ensures each batch meets safety, efficacy and quality standards (Lener et al. 2015).

### 4.2 General QC Requirements in Manufacture of EV Products

#### 4.2.1 QC at the Production Stage

EV production should ideally use xenogeneic‐free, serum‐free culture media where possible to avoid contamination with potentially hazardous agents. For example, foetal bovine serum (FBS) may contain contaminants such as EVs, mycoplasma, viruses and endotoxins, as well as xenogeneic proteins and lipids that could be incorporated into EVs. Regulatory guidelines recommend using media cleared of bovine EVs through ultrafiltration (Kornilov et al. 2018), heat inactivation at 56°C for 30–60 min to destroy complement activity and inactivate potential microbial contaminants (Urzì et al. 2022) or chemically defined serum substitutes to minimise impurities and variability (Usta et al. 2014). Such treatment might have adverse effects such as reduced cell attachment, proliferation and cell metabolic activities, which need to be considered on a case‐by‐case basis (Mochizuki and Nakahara 2018). Key production parameters such as cell seeding density, concentration, pH and oxygen levels must be tightly controlled to ensure EV yield and quality. The quantity of these parameters can be variable based on the bioreactor and cell type. For instance, a cell seeding density of 3000–6000 cells/cm^2^ and concentration of 1 × 10^5^ cells/mL have been reported for the adherent culture of MSCs in microcarriers (De Sousa Pinto et al. 2019; De Almeida Fuzeta et al. 2020). The dissolved oxygen levels should be adjusted according to the application of EVs. For instance, a recent study showed hypoxia (5% O_2_) conditioned MSC‐derived EVs increased vascular tube formation (Almeria et al. 2019). The number of EVs produced by cells depends on the type of cell and the conditions under which they are cultured. A total of 2000 and 3500 EVs/cell were reported for adipose‐derived stem cells (ADSCs) and Wharton's jelly‐MSCs (Tsai et al. 2022), while a value of 10^4^–10^5^ EVs/cell was reported for MSCs and neurons (Karttunen et al. 2022).

#### 4.2.2 QC Postproduction

After production, characterisation focuses on defining EV attributes, properties and functional testing to verify that EVs meet the specified target product profile. Consistent size and morphology are critical for EV function and pharmacokinetics. Ideally, EVs for therapeutic applications should range in size from 50 to 200 nm, with size variability under 20% per batch to ensure consistency. Size analysis via nanoparticle tracking analysis (NTA) or dynamic light scattering (DLS) should yield a PDI below 0.2, while PDIs above 0.3 suggest high size heterogeneity and may fail QC standards (Danaei et al. 2018). Morphologically, EVs should maintain a round or cup shape as observed by cryo‐electron microscopy (cryo‐EM) (Chuo et al. 2018), since deviations can potentially lead to reduced cellular uptake and alteration in EVs therapeutic function.

Tetraspanins CD63, CD9 and CD81 are standard markers for confirming typical EV surface protein expression (Andreu and Yáñez‐Mó 2014), while luminal markers such as TSG101 and ALIX indicate endosomal origin (Falguières et al. 2008). In regenerative medicine, MSC‐derived EVs should also contain specific proteins like Annexin A1 and CD73, for inflammation modulation (Conrad et al. 2019). QC requires verifying these protein levels using Western blotting or nanoflow cytometry (Ngo et al. 2025), aiming for target expression within a ±10% range of reference standards (Geeurickx et al. 2021). In MSC‐derived EVs, QC should verify the presence of miRNAs such as miR‐21 and miR‐146a, which are involved in wound healing and antiinflammatory pathways (Jiang et al. 2022). For lipids, specific sphingomyelins and phosphatidylserines should meet target concentrations to support EV stability and bioactivity (Haraszti et al. 2016). QC should confirm a minimum RNA content of femtograms to picograms depending on the cell type and therapeutic applications (Middleton et al. 2018).

Purity is evaluated by the particle‐to‐protein ratio, with an acceptable benchmark often set at 10^10^ particles per µg protein to minimise protein contamination (Webber and Clayton 2013). Impurities from cell culture, like residual bovine albumin, should be minimised to meet clinical standards. Pathogen safety is especially crucial for EVs derived through viral transfection. Regulatory guidelines require that viral load in therapeutic EV products is less than 10^4^ copies/mL (Guideline on the Clinical Evaluation of Medicinal Products intended for Treatment of Hepatitis B 2006), while testing for common pathogens (e.g., mycoplasma, endotoxins) should yield nondetectable levels, ideally under 0.2 EU/mL for endotoxin (https://www.fda.gov/regulatory‐information/search‐fda‐guidance‐documents/endotoxin‐testing‐recommendations‐single‐use‐intraocular‐ophthalmic‐devices).

QC should also assess EV uptake efficiency in target cells, typically using fluorescence or radiolabelling techniques. MSC‐derived EVs for wound healing, for instance, should exhibit over 70% uptake in target cells within 24 h in vitro (Fuhrmann et al. 2015). This helps ensure that EVs will be effectively internalised for therapeutic activity *in vivo*. Therapeutic EVs should demonstrate a specific bioactivity aligned with their intended Mechanism of Action (MoA). In regenerative therapies, MSC‐EVs should show an increase in cell proliferation assays (Vonk et al. 2018) compared to controls, while immune‐modulating EVs may require cytokine secretion upregulation or inhibition in relevant immune cell models (Mahmoudi et al. 2023). Stability tests should ensure that EVs retain their bioactivity and size integrity for at least 6 months at −80°C, with a particle loss of less than 20%. In vivo, pharmacokinetic studies typically aim for EV circulation half‐lives of 2–6 h post‐administration, depending on the application (Kang et al. 2021). For clinical relevance, EVs should demonstrate consistent biodistribution and therapeutic effects in target tissues with minimal off‐target effects.

### 4.3 The Role of Microfluidics in Quality Control of EV Products

Microfluidics can be crucial in assessing the quality of EV products, leading to high levels of consistency and reproducibility in alignment with GMP standards (Piffoux et al. 2022). Table 4 provides a comparative overview of microfluidic technologies that can be applied for EV QC, highlighting analytical targets, detection principles, sensitivity and suitability for potency testing, molecular profiling and in‐line manufacturing integration. These platforms span from digital RT‐PCR and chemiluminescent assays to super‐resolution imaging and sequencing, offering a toolkit for both high‐throughput batch assessment and functional validation at the single‐EV or single‐cell level.

**TABLE 4:** Microfluidic platforms for EV quality control and characterisation.

Platform/study	Sample type	Characterisation target	Detection mode/principle	Limit of detection (LOD)	Multiplexing capability	Real‐time monitoring	Resolution/sensitivity	Throughput	Application	Ref
fluoMDS microfluidic diffusion sizing	MSCs, HEK293F, microalgae EVs	Size, concentration, lipid/protein content, CD63	Lateral diffusion + multiwavelength fluorescence imaging	Not explicitly stated	Yes—via multichannel fluorescence	Yes	∼50–200 nm size resolution; subpopulations and impurities	Minutes/sample with <5 µL	Rapid multiparametric screening	(Paganini et al. 2022)
SEA‐chip (Single EV Analysis)	Gli36‐derived EVs (WT, EGFRvIII, IDH1R132H)	Panel of 11 Protein markers (e.g., CD63, EGFR, IDH1)	Fluorescence microscopy with immunostaining	∼10⁷ EV/mL	Yes—up to 11 protein markers	No	Single EV resolution; heterogeneity and rare subpopulations	High‐content imaging (>1000 EVs per image in <1 s)	Detailed EV immunoprofiling from small volumes	(Lee et al. 2018)
Microfluidic Chemiluminescent ELISA	Cell culture medium (bladder, breast cancer)	CD9 (quantification), EGFR, HER2, MHC‐I, EpCAM	Immunoprecipitation + sandwich chemiluminescent ELISA	8.7 × 10⁷ EVs/mL	Yes (4–5 proteins)	No	Linear range >3 logs; sensitive to <40 ng input protein	<1 h per sample; ∼8 µL input	Label‐free, direct EV quantification from unpurified medium	(Tan et al. 2021)
PS‐ED (Plasma Separation–EV Detection) chip	Whole blood	EV concentration; CD81, CD24, EpCAM	Chemiluminescence‐based immunoassay (CL‐ELISA)	95 particles/µL	Yes (3 markers)	No	Linear range: 2.5 × 10^2^ to 2.5 × 10⁸ particles/µL	78 min per sample (total workflow)	Clinical biomarker validation from complex fluids	(Zhou et al. 2020)
AC‐EHD nanoshearing Colorimetric Chip	Breast and prostate cancer cell culture	CD9, HER2, PSA	AC electrohydrodynamic‐induced nanoshearing + colorimetric ELISA (TMB/HRP)	2760 exosomes/µL	Yes (up to 3 markers)	No	∼3× sensitivity improvement vs. pressure‐driven flow	∼2 h per run; manual readout	Rapid and selective exosome profiling for multiplexed QC	(Vaidyanathan et al. 2014)
SERS‐Integrated Microfluidic Chip	LNCaP, PrEC cell‐derived EVs; Serum	CD63, EpCAM	Sandwich immunoassay + Raman spectroscopy	1.6 × 10^2^ particles/mL	No	No	Linear 10^2^–10⁹ particles/mL; ∼77% capture efficiency	∼1 h per sample; 20 µL input	Detection of low‐abundance targets in clinical EVs	(Wang et al. 2020)
seiSEQ (single‐EV immunosequencing)	Gli36 EVs, RAW264.7 macrophage EVs	CD9, CD63, CD81, EGFR, CD45, CD11b, F4/80	DNA‐barcoded antibody labelling + droplet microfluidics + next‐gen sequencing	Not directly reported; detects femtomolar targets	Yes (≥8 markers tested)	No	Single‐EV resolution; highly specific barcoding, minimal crosstalk	∼1100 EVs/sample with 50,000 reads	Multiplexed profiling of EV protein heterogeneity for QC	(Ko et al. 2021)
Droplet Microfluidic One‐Step RT‐PCR	Plasma from lung cancer patients; synthetic miR‐21‐5p	miR‐21‐5p (exosomal microRNA)	One‐step reverse transcription PCR in droplets	Single miRNA molecule per droplet	No	No	Detects ∼1 copy per 82.5 pL droplet; ∼45% signal at *λ* = 1	∼µL input; ∼thousands of droplets/run)	Sensitive RNA‐level QC; applicable to potency testing	(Cui et al. 2020)
Optical Nanopore Quantification (ZMW‐based)	EVs and virus‐like particles (HIV, AAV, MLV, HBV, etc.)	Concentration and size via translocation	Zero‐mode waveguide (ZMW) fluorescence + hydrodynamic nanopore translocation	10⁵ particles/mL in 60 s; ∼10^3^ particles/mL in extended 1 h read	Yes (via fluorophore‐specific detection)	Yes	Single‐particle resolution; <4% error in concentration across 6 logs	High (10⁵ pores in parallel; <15 min total assay)	QC of EV/virus mixtures in low volume	(Chazot‐Franguiadakis et al. 2022)
Digital mRNA assay chip (dual‐probe hybridisation)	EV lysates from CHLA‐9, CHLA‐258 (PNET cell lines)	GAPDH and EWS‐FLI1 mRNA (tumour‐associated)	PCR‐free dual‐probe hybridisation and enzymatic fluorescence in femtoliter wells	18 aM for synthetic GAPDH mRNA	No	No	Single‐molecule detection; 0.277–7.25 copies per 10⁵ EVs	12,000 microwells per chip; multiple parallel units possible	High‐sensitivity transcript quantification in EVs for mutation screening	(Zhang et al. 2018)
Coculture diffusion chip with hydrogel barriers	U937 cells; exosomes labelled with Cy5 or GFP	EV secretion, diffusion, uptake by recipient cells	Fluorescent imaging (Cy5 and GFP) of labelled EVs	N/A	No	Yes (with live imaging capability)	Visual confirmation of EV uptake; qualitative resolution	∼2–3 days per experiment; multi‐timepoint imaging	Functional potency assay simulating physiologic EV exchange	(Mason et al. 2022)
3D Microfluidic Chip for EV Internalisation	ASC‐derived EVs in articular chondrocytes and fibroblast‐like synoviocytes	EV‐cell interaction: binding vs. internalisation	Confocal microscopy and colocalisation image analysis	N/A	No	Yes (multi‐timepoint imaging)	Single‐cell, single‐EV spatial discrimination in 3D	∼24 h interaction window	Potency testing; validating EV uptake and extracellular matrix interaction in near‐physiological conditions	(Ragni et al. 2020)
Dual‐fluorescence droplet array chip	Huh‐7‐CD9‐mCherry cells; purified EVs	Simultaneous secretion of Matrix Metalloproteinases (MMPs) and CD9+ EVs at single‐cell level	Droplet‐based microfluidics with time‐lapse fluorescent imaging (MMP‐GFP + CD9‐mCherry)	∼10^3^ EVs/droplet (∼330 pL volume)	No	Yes (time‐lapse fluorescence every 3 h)	Single‐cell temporal resolution; subpopulation shifts in EV and MMP release	∼3000 droplets per chip; ∼12 h time‐lapse window	Functional potency test linking vesicle subtype to bioactivity (MMP secretion)	(Ji et al. 2025)
Superresolution Flow and Pdot‐Based Imaging	Seminal exosomes	CD9, CD63, CD81 copy number and spatial mapping	3‐Colour superresolution imaging using photoswitchable Pdots	Single tetraspanin per exosome (<5 nm localisation error)	Yes (CD9, CD63, CD81)	No	<5 nm localisation precision; single‐EV protein counting	∼1000 exosomes/s (flow); hundreds imaged in 5 min	Quantification of EV protein copy number and spatial organisation	(Jiang et al. 2021)

#### 4.3.1 Microfluidic QC in the Production Stage

**FIGURE 6:**
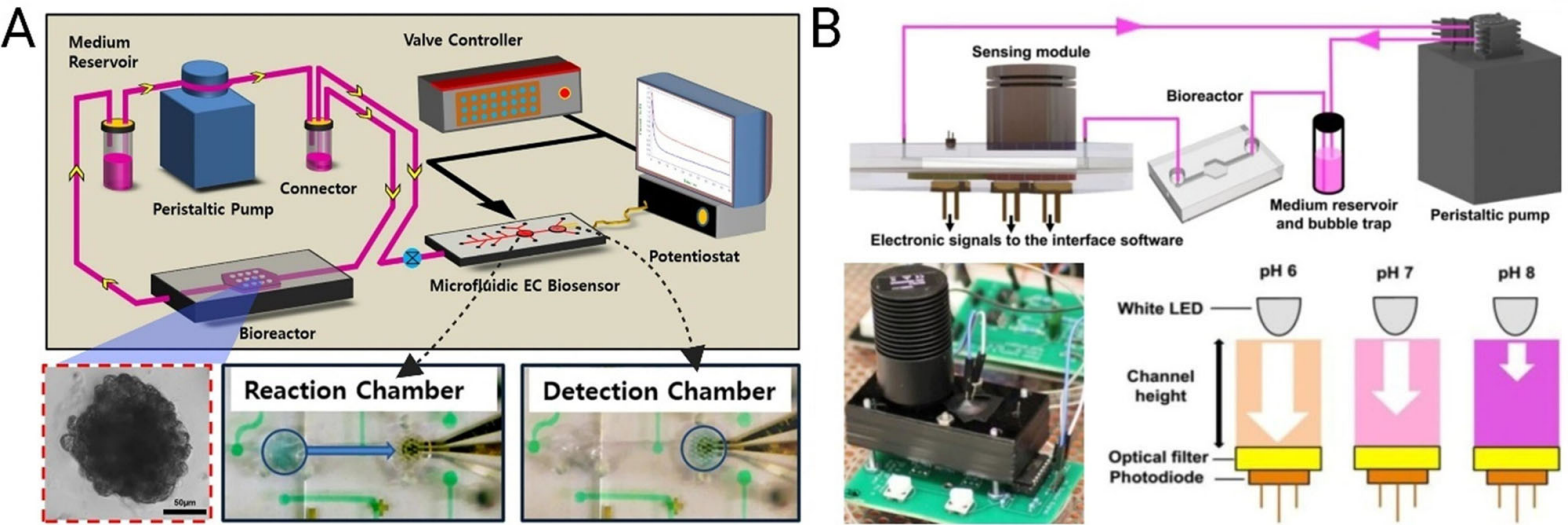
Microfluidics for quality control assessment of manufactured extracellular vesicles (EVs) at the production stage. (A) A Microfluidic electrochemical sensor for monitoring metabolic activity of hepatocyte cultures under acetaminophen (APAP) administration, reproduced from (Riahi et al. 2016) under Creative Commons Attribution 4.0 International License; (B) A multianalyte pH and oxygen system integrated with a microfluidic bioreactor sensor, reproduced from (Mousavi Shaegh et al. 2016) with permission from AIP publishing.

Due to their compact design, high surface to volume ratio and excellent precision manufacturability, a wide range of sensors can be integrated into microfluidic bioreactors to measure culture conditions such as nutrient availability, cell metabolic activity, pH and dissolved oxygen levels and pathogen levels (Buttkewitz et al. 2023; Bunge et al. 2019). This enables precise control of the culture conditions for EV‐producing cells, enhancing reproducibility in EV size distribution, yield and composition (Piffoux et al. 2017). For instance, a microfluidic electrochemical immunosensor platform has demonstrated the ability to continuously monitor cell‐secreted biomarkers (Figure 6A) (Riahi et al. 2016). Transferrin and albumin secretion in hepatocyte cultures under different acetaminophen (APAP) concentrations was monitored in real‐time with high sensitivity (0.2 and 0.5 ng/mL for transferrin and albumin, respectively) and long‐term functionality (over 7 days culture) without the limitations of conventional methods such as ELISA or mass spectrometry (MS). Presented in Figure 6B, Mousavi Shaegh et al. (2016) introduced an optical multianalyte sensing module capable of real‐time dynamic measurement of pH and oxygen levels in microfluidic bioreactors. Their system achieved high sensitivity for pH (160 mV/pH) and oxygen (6 mV/%O_2_) monitoring with minimal noise, supporting long‐term cell culture processes.

#### 4.3.2 Biophysical and Biochemical Characterisation Using Microfluidics

- Size and Morphology Analysis

**FIGURE 7:**
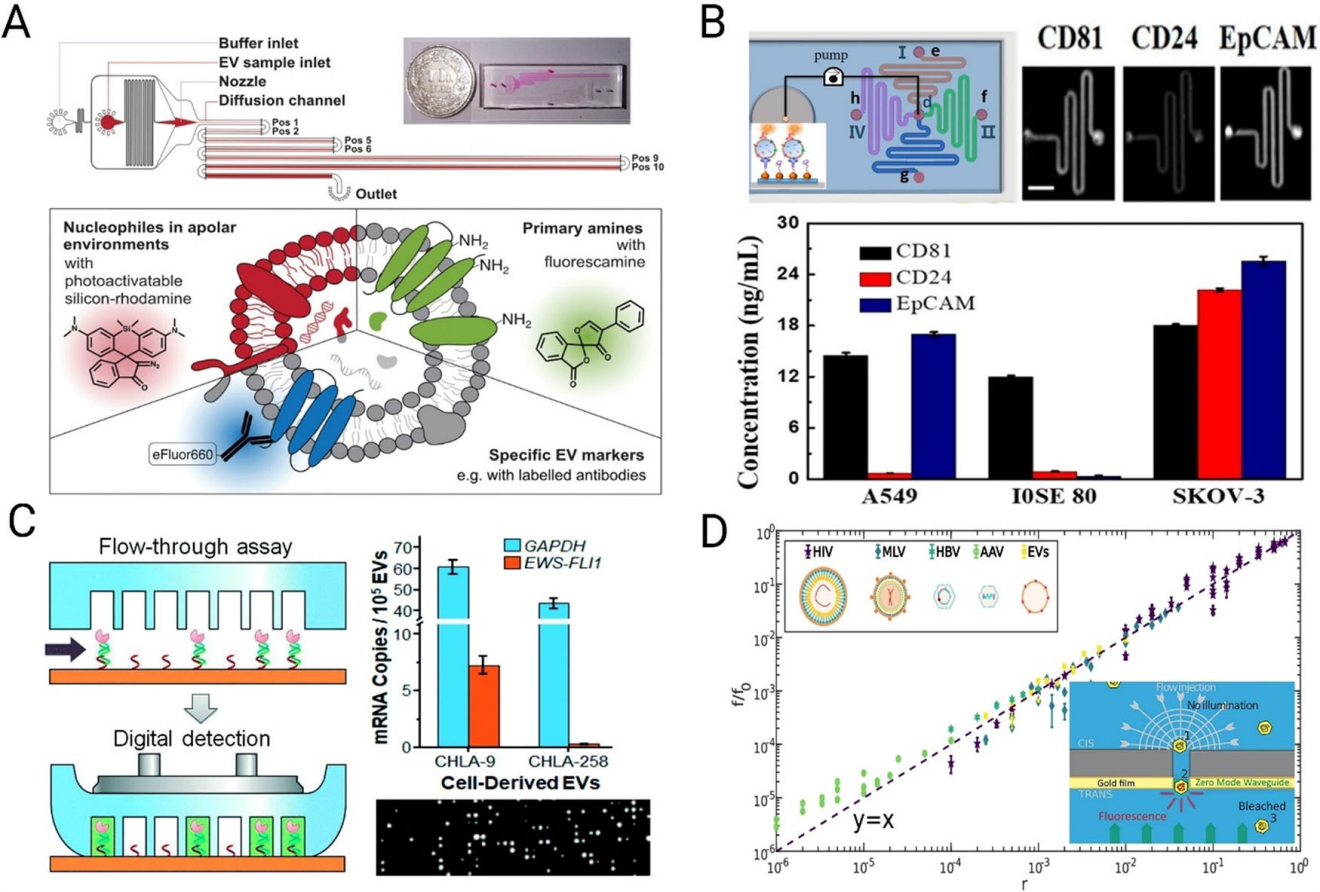
Biophysical and biochemical quality control (QC) of produced extracellular vesicles (EVs) using microfluidics (A) fluorescence‐based microfluidic diffusion sizing (fluoMDS) enabling EV size, morphology and molecular profiling, reproduced from (Paganini et al. 2022) with permission from John Wiley and Sons; (B) Microfluidic immunoassays for multiplex EV protein biomarker analysis (here CD81, CD24 and EpCAM from three different cell lines)‐inset showing sandwich assay, reproduced from (Zhou et al. 2020) with permission from American Chemical Society; (C) Microwell‐patterned microfluidic digital analysis chip for PCR‐free detection of cell culture derived EV mRNAs of GAPDH and EWS‐FLI1 Type 1; black and white inset image shows fluorescent image of detecting microwells, reproduced from (Zhang et al. 2018) with permission from the Royal Society of Chemistry. (D) Pathogen detection and viral safety measurement using hydrodynamic nanopore system. A wide range of viruses (HIV, MLV, HBV and AAV) as wells as EVs were detected based on their translocation frequency through the nanopores, reproduced from (Chazot‐Franguiadakis et al. 2022), with permission from the American Chemical Society.

Microfluidic NTA platforms can measure EV sizes with accuracy within ±10 nm (Longjohn and Christian 2022). Paganini et al. (2022) introduced fluorescence‐based microfluidic diffusion sizing (fluoMDS), a novel approach to EV characterisation that addresses key limitations of conventional methods like NTA. Unlike NTA, which is biased by larger particles and requires substantial time and sample volume, fluoMDS enables rapid, multiparametric analysis of EV size, concentration and composition using only a few microlitres of sample (Figure 7A). Operating directly in solution, it eliminates the need for immobilisation or extensive washing steps. By integrating specific and unspecific fluorescent labelling, fluoMDS accurately identifies EV subpopulations and contaminants, making it a powerful tool for purity assessment and bioprocess optimisation.

Microfluidic platforms also offer significant advantages for EV morphological analysis using cryo‐EM by providing precise control over sample preparation (Fuest et al. 2019). Microfluidics can regulate sample flow to ensure uniform and rapid freezing of EVs in their native hydrated state, a critical step for achieving high‐resolution cryo‐EM imaging (Mäeots et al. 2020). Additionally, parameters such as shear stress, temperature and flow rate can be finely tuned within microfluidic channels, preserving EV morphology and preventing structural alterations during preparation. This approach reduces dehydration and artefacts often associated with conventional cryo‐EM sample preparation methods, enabling the visualisation of EVs in their natural state with greater accuracy and reliability.

- Protein Marker Identification

Microfluidic platforms enable sensitive and multiplexed analysis of EV surface proteins, supporting batch consistency and potency assessment during therapeutic manufacturing. Lee et al. (2018) developed the SEA platform for single‐vesicle fluorescence profiling of markers such as CD9, CD63, CD81, EGFR and IDH1, achieving high‐throughput and sub‐second imaging. Tan et al. (2021) introduced a chemiluminescent ELISA chip that quantified CD9 and profiled multiple functional markers (e.g., EGFR, HER2) from unpurified EVs with high sensitivity using only ∼8 µL of input. Ko et al.’s (2021) *seiSEQ* platform used DNA barcoding and droplet microfluidics for highly multiplexed, sequencing‐based protein profiling of individual EVs. A single microfluidic chip with four channels, each modified with a unique capture antibody, has been used for multiplex biomarker analysis of cell culture‐derived EVs (Figure 7B) (Zhou et al. 2020). A key advantage of microfluidic protein biomarker analysis is the minimal sample volume required; these immunoassays can detect protein markers in as little as 10 µL of sample.

- Molecular Cargo Detection

**FIGURE 8:**
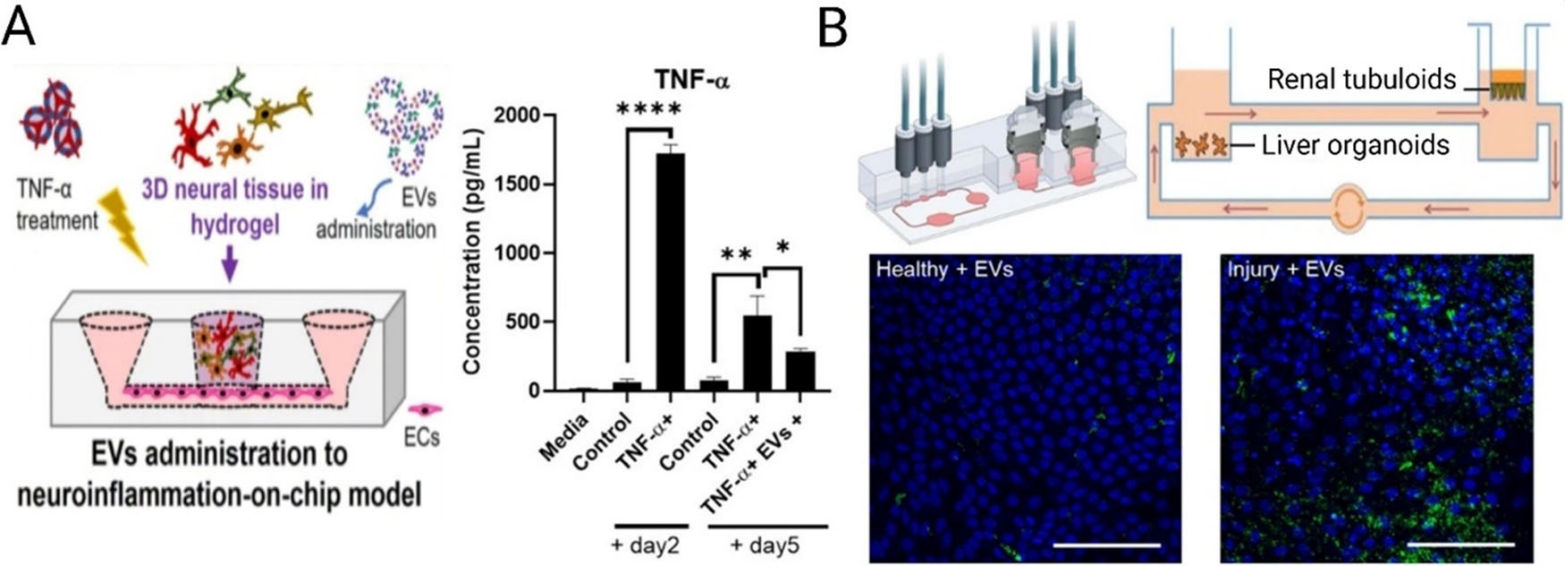
Functional characterisation of manufactured extracellular vesicles (EVs) using microfluidics: (A) Therapeutic potency and mechanism of action using organs on chip disease models – here mesenchymal stromal cell (MSC) derived EVs were evaluated for neural cell differentiation and neural tissue formation, antiinflammatory effect of MSC‐derived EVs is shown in reduction in tumour necrosis factor‐alpha (TNF‐α) secretion, reproduced from (Saglam‐Metiner et al. 2024) with permission from Springer Nature and (B) Multiorgan microfluidic models of EV circulation and accumulation integrating kidney and liver organoids; MSC‐derived EVs selectively accumulated in injured kidney tissue, reproduced from (Nguyen et al. 2022) under Creative Commons CC BY license.

Microfluidic devices facilitate the analysis of EV molecular cargo, including RNA and lipid content. For example, microfluidic quantitative PCR (qPCR) systems can detect microRNAs such as miR‐21 and hsa‐miR‐21‐5p within EVs, with a detection limit as low as 20 fM (Cui et al. 2020). Microfluidic methods for liquid biopsies have also been utilised to analyse tumour mRNAs isolated from EVs using a microwell‐patterned microfluidic digital analysis chip for PCR‐free detection (Zhang et al. 2018) (Figure 8C). The platform integrates mRNA capture, tagging and single‐molecule digital detection, achieving an ultrasensitive limit of detection (LOD) as low as 20 aM. This microfluidic digital assay chip allowed for the quantification of the levels of *GAPDH* and *EWS‐FLI1* Type 1 mRNA transcripts in CHLA‐9 and CHLA‐258 derived EVs (Figure 8C), but could be applied to other mRNA transcripts of interest.

- Purity and Non‐EV Component Removal

Microfluidic devices can be used as a QC for measuring EVs purity from contaminants and non‐EV particles. For instance, the fluorMDS system enables robust measurement of EV purity by integrating size distribution analysis with fluorescence‐based identification of specific biomarkers and contaminants (Paganini et al. 2022). By distinguishing particles containing lipids, primary amines and EV markers (e.g., CD63), the technique identifies and quantifies impurities such as protein and lipid aggregates. For example, fluoMDS detected a significant presence of primary amine‐rich contaminants in impure HEK293F‐derived EV samples, with an average diameter of 19 nm, correlating with reduced particle‐to‐protein ratios and higher contaminant levels compared to purified MSC‐derived EVs. This capability facilitates accurate EV purity assessments critical for bioprocess optimisation.

- Pathogen Removal and Viral Safety

Using nanofilters with pore sizes between 20 and 50 nm, microfluidic systems can reduce viral load to clinically acceptable levels (below 10^4^ copies/mL). Integration of sensing platforms into microfluidic nanopore systems can also allow quantification of pathogenic and viral loads in EV products. As presented in Figure 7D, Chazot‐Franguiadakis et al. (2022) demonstrated a nanopore‐based optical sensing platform capable of distinguishing EVs from viral particles with a detection limit of 10^3^ particles/mL for viral pathogens after 1 h of acquisition. This system uses fluorescent labelling to differentiate EVs and viral particles based on their unique translocation signals, enabling precise concentration measurements and effective QC in EV‐related applications.

#### 4.3.3 Functional Characterisation of EVs Using Microfluidics

Microfluidic platforms have been developed for functional testing of EVs by several different tests. Functional testing on microfluidic platforms provides detailed insights into the bioactivity of EVs, a crucial aspect of QC.

- Cellular Uptake and Targeting Efficiency

Microfluidic platforms have emerged as useful tools for studying EV targeting efficiency and cellular uptake, enabling precise and high‐throughput analysis. For instance, Mason et al. (2022) developed a microfluidic platform to monitor real‐time EV exchange between co‐cultured cells using selectively permeable barriers that mimic physiological conditions. Their system allowed for detailed tracking of EV uptake dynamics, across the barrier, highlighting EV targeting specificity and functional engagement with cells. Ragni et al. (2020) utilised microfluidics combined with 3D confocal microscopy to assess the uptake of MSC‐derived EVs by fibroblast‐like synoviocytes and articular chondrocytes in osteoarthritis models, finding that EV uptake was enhanced in a 3D microenvironment compared to 2D cultures, emphasising the role of extracellular matrix components in EV internalisation. Ji et al. (2025) developed a droplet‐based microfluidic system for single‐cell analysis, revealing a real‐time correlation between EV secretion and cellular responses, further refining the understanding of EV‐mediated communication. These platforms demonstrate the power of microfluidics to quantify EV uptake, often achieving high precision with minimal sample loss, and support real‐time tracking to ensure functional and targeting specificity.

- Therapeutic Potency and Mechanism of Action (MoA)

Microfluidic platforms provide a powerful approach for assessing the therapeutic potency and MoA of EVs in various disease contexts, including cardiac repair, neuroinflammation and cancer. Organ‐on‐chip models such as cardiac tissues‐on‐a‐chip demonstrated that EVs could significantly enhance cardiomyocyte proliferation and angiogenesis, with studies reporting a 45% increase in angiogenic sprouting within 24 h of EV treatment (Wagner and Radisic 2021). Similarly, neuroinflammation models (Figure 8A) using neural tissue‐on‐chip systems have shown that MSC‐derived EVs reduce tumour necrosis factor‐alpha (TNF‐α)‐induced cytokine secretion by 60%, highlighting their immunomodulatory effects (Saglam‐Metiner et al. 2024). In cancer models, the bioactivity of tumour cell‐derived EVs was quantified using microfluidic tumour microenvironment mimics, revealing over 70% suppression of stromal cell migration in the presence of targeted therapeutic EVs (Ural et al. 2021). These platforms also enable precise monitoring of cytokine secretion, cell migration and differentiation, providing high‐throughput, reproducible assays for therapeutic evaluation while maintaining physiological relevance.

- In‐Vivo Kinetics

Microfluidic systems offer a robust platform for studying the behaviour of EVs, providing insights into their potential biodistribution and pharmacokinetics in vivo. Nguyen et al. (2022) demonstrated a multiorgan‐on‐a‐chip model integrating kidney and liver organoids (Figure 8B); MSC‐derived EVs selectively accumulated in injured kidney tissue, mirroring *in vivo* biodistribution. Similarly, Ronaldson‐Bouchard et al. (2022) utilised a vascularised multitissue chip showing CD63 sEVs secreted by heart tissues were distributed across all tissues after two weeks of culture, with immunofluorescence confirming sEV uptake by endothelial cells beneath the heart tissue. These systems replicate physiological environments with controlled flow and shear stress, enabling precise and high‐throughput evaluation of EV biodistribution and therapeutic potential without reliance on animal models.

### 4.4 Coupling Microfluidics With Analytical Systems

Microfluidic platforms can be coupled with established analytical systems like flow cytometry, MS and next‐generation sequencing (NGS) to enhance EV QC and characterisation capabilities. Coupling microfluidics with flow cytometry overcomes limitations of conventional flow cytometry, which struggles to detect EVs below 100 nm. Microfluidic systems enable single‐EV analysis by optimising flow conditions and employing advanced detection methods such as fluorescence, impedance and imaging‐based techniques. Van Der Vlist et al. (2012) modified a high‐resolution flow cytometer to detect and quantify fluorescently labelled EVs, achieving precise identification of EV subpopulations based on membrane protein composition. Akagi et al. (2015) combined microfluidic‐controlled flow together with single‐molecule‐sensitive detection and super‐resolution imaging to map tetraspanin protein expression on EVs at 100 EVs/s. This approach facilitates high‐throughput, reproducible analyses, including multiparametric characterisation of EV subpopulations, supporting the standardisation of EV‐based therapeutics. Such advancements ensure accurate monitoring of critical quality attributes like size, protein expression and bioactivity, vital for clinical applications. Coupling microfluidics with MS enhances pre‐concentration, separation and delivery to MS devices while enabling detailed profiling of EV contents such as lipids and signalling proteins. Lazar et al. (2020) highlighted that microfluidics integrated with nano‐electrospray ionisation enables the detection of low‐abundance proteins from EVs, achieving detection limits in the low attomole range, ideal for EV proteomics and lipidomics. This microfluidic‐MS system identified thousands of EV‐associated proteins and lipids, providing insights into their molecular composition and posttranslational modifications (Kreimer et al. 2015). Coupling microfluidics with NGS enhances EV characterisation by enabling efficient, high‐throughput analysis of their nucleic acid cargo. Droplet‐based microfluidics precisely encapsulates and barcodes EV‐derived nucleic acids, minimising contamination and amplification bias. For instance, this method was used to develop a microfluidic device for DNA size selection in NGS library preparation, demonstrating high reproducibility with a carryover rate of less than 3.5% and full compatibility with downstream sequencing (Serra et al. 2020).

## 5 Challenges and Future Perspectives

Microfluidics promises to enhance the precision and scalability of the many processes required in therapeutic EV production and analytics. However, several technical and regulatory challenges remain, particularly in achieving reliable, scalable, and sterile EV production. Below, we delve into key technical obstacles, the need for combined isolation methods, microfluidic impact on commercialisation, emerging innovations and standardisation.

### 5.1 Technical Challenges in EV Production

Microfluidic systems present unique technical issues that must be addressed to ensure high‐quality therapeutic EV production at a clinical scale.

#### 5.1.1 Clogging and Fouling

Clogging and fouling represent significant technical challenges in microfluidic EV manufacturing, adversely impacting system efficiency and EV yield. These phenomena are primarily induced by the accumulation of particles or biomolecules, such as proteins and lipids, on channel walls or filtration membranes. Factors like particle aggregation, high shear stress and inadequate cleaning protocols exacerbate the issue (Wang et al. 2024; Chernyshev et al. 2023). Detection of clogging and fouling typically involves monitoring pressure drop across membranes or reduced flow rates in the microfluidic channels. For example, a filtration‐based microfluidic device showed fouling when filtering EVs from serum samples due to protein and lipid buildup, leading to diminished filtration efficiency over time (Inci 2022). Pressure drops of 104.6 kPa for 50 nm pore filters when operating at 60 µL/min highlight how clogging impacts system performance. Such issues can significantly disrupt the manufacturing process, reducing EV yield and purity while increasing operational costs and process downtime. To mitigate these effects, strategies like introducing herringbone grooves for enhanced mixing (Huang et al. 2023), applying hydrophobic or antifouling coatings on channel surfaces (Zhang et al. 2016) can be employed. Filtration systems can also maintain operational efficiency over multiple runs. For instance, a cascaded microfluidic circuit employing pulsatile filtration demonstrated clog‐free and high‐purity isolation of EVs from blood (Li et al. 2023). Despite these advancements, these challenges remain for clinical scalability.

#### 5.1.2 Material, Geometry and Flow‐Induced Stressors in Microfluidic EV Systems

Although microfluidic systems have demonstrated powerful capabilities for EV purification, concerns about how substrate materials, channel geometries and flow regimes may affect the structural and functional integrity of EVs needs more investigation. From a production standpoint, the composition and mechanics of microfluidic materials, commonly PDMS, glass or thermoplastics, can influence EV adsorption, recovery and potential leaching of uncured monomers (Hirama et al. 2021). Furthermore, complex flow dynamics, such as those found in serpentine channels or oscillatory acoustics, may alter membrane fluidity, curvature or even cargo retention of EVs (Thompson and Papoutsakis 2023).

These effects have raised valid concerns about how engineered shear stress, surface interactions and extended residence times may impact protein, RNA or lipid cargo. Nevertheless, strategies such as surface passivation [e.g., with a phospholipid copolymer containing 2‐methacryloyloxyethyl phosphorylcholine and 3‐methacryloxyethyl triethoxysilane (Akagi et al. 2015)] and low‐shear design optimisation (Delon et al. 2020) have been developed to mitigate such risks. Advances in inertial‐elastic focusing (Meng et al. 2023), hybrid acoustofluidics (Wang et al. 2023) and the use of fluorinated ethylene propylene or cyclic olefin copolymer chips (Agha et al. 2022) further improve EV compatibility, while enabling scalability.

#### 5.1.3 Release of Captured EVs

Controlled and complete release of EVs following immunoaffinity capture is a significant technical challenge in microfluidic EV manufacturing, as it impacts both yield and EV integrity. Immunoaffinity‐based methods employ antibodies targeting surface markers like CD63, CD81 or EpCAM to isolate EVs with high specificity; however, the strong antigen‐antibody interactions can impede the gentle release of intact EVs for downstream applications (Hisey et al. 2018; Lo et al. 2020). For instance, Lo et al. utilised a radial‐flow microfluidic chip modified with biotin‐avidin chemistry for EV capture but overcame the irreversibility issue by incorporating desthiobiotin‐conjugated antibodies. This enabled efficient EV release under mild elution conditions, preserving their integrity and functionality (Lo et al. 2020). Similarly, Dehghani et al. demonstrated tangential flow microfluidics (TFM) for capturing and releasing EVs using ultrathin membranes. TFM successfully reduced membrane fouling and allowed EV release under minimal TMPs, maintaining up to 90% EV recovery efficiency. Another study by Hisey et al. employed low pH buffers to disrupt antibody–antigen interactions in a herringbone‐grooved microfluidic device, facilitating the release of intact and label‐free EVs while ensuring stability through rapid neutralisation (Dehghani et al. 2019). These systems preserve EV structural and functional integrity while maintaining yields crucial for therapeutic manufacturing. Future advancements must focus on optimising release conditions, such as exploring cleavable linkers, peptide‐based affinity systems, or external stimuli like light or temperature, to further enhance clinical scalability (Suwatthanarak et al. 2021; Lewis et al. 2018). Additionally, the integration of automated, high‐throughput microfluidic systems with gentle and efficient EV release mechanisms is critical for achieving GMP compliance and clinical viability.

#### 5.1.4 Production Consistency

Controlling shear stress is a critical but challenging aspect of consistent EV production in microfluidic bioreactors. Shear stress directly influences the behaviour of cells, affecting EV secretion, size distribution and bioactive content. Studies by Kang et al. (2022) revealed that optimal shear stress levels between 10⁻⁴ and 10⁻^3^ dyn/cm^2^ enhanced MSC viability and EV production, with yields up to seven times higher compared to static culture conditions. Conversely, excessive shear stress, such as levels exceeding 8.3 dyn/cm^2^, can induce abnormal cell morphology, disrupt cell attachment and compromise EV quality (Yu et al. 2024). Achieving consistent control over shear stress is challenging due to its dependence on flow rate, channel geometry and cell adhesion dynamics. Variations in these parameters can lead to inconsistent EV yield and functional properties. Active modulation of shear stress using programmable pumps or shear‐sensing feedback systems has been explored, enabling dynamic control of stress levels between 0.4 and 15 dyn/cm^2^ (Yu et al. 2024; Kang et al. 2022). However, implementing these strategies requires precise engineering and real‐time monitoring to maintain reproducibility across production batches. The challenge is further compounded by the need to standardise shear stress levels across different cell types, as cellular responses to stress can vary.

Automation and real‐time monitoring in microfluidic bioreactors remain critical challenges for consistent EV production. Advanced sensor‐integrated microfluidic platforms, such as those reported by Mousavi Shaegh et al. (2016), enable real‐time monitoring of key parameters like pH and oxygen, ensuring a stable microenvironment essential for reproducible EV quality. Similarly, Foscarini et al. (2024) developed lab‐on‐chip systems incorporating capacitive sensors to detect cell density changes, which could be adapted for monitoring EV concentration and size distribution during production. Despite these advancements, further work is needed to miniaturise sensors and improve their integration with microfluidic systems to prevent issues like leakage or biofouling, as highlighted by Buttkewitz et al. (2023). Future developments in sensor integration, coupled with AI‐driven analytics, could enable fully automated, scalable production workflows for EV manufacturing, meeting regulatory standards and facilitating clinical translation.

#### 5.1.5 Scaling Up While Maintaining Sterility

Scaling up microfluidic systems for clinical‐grade EV production introduces significant challenges in maintaining sterility. Microfluidic devices, especially those made of PDMS or polymeric materials, have narrow channels that are difficult to sterilise, leading to risks of contamination. Pathogen exposure, endotoxin accumulation or material‐induced toxicity can compromise EV integrity and therapeutic efficacy (Varma and Voldman 2018). Increased surface‐to‐volume ratios in miniaturised devices can promote fouling and biofilm formation, further exacerbating sterility issues (Mukhopadhyay 2005). Pathogen contamination not only disrupts production but can also trigger inflammatory responses in downstream clinical applications (Varma and Voldman 2018).

Effective sterilisation methods include autoclaving, UV exposure, ethylene oxide (EtO) and hydrogen peroxide (H_2_O_2_) treatments, each with specific trade‐offs. Autoclaving at 121°C for 20 min damages heat‐sensitive polymers like PMMA, altering surface morphology and transparency (Yavuz et al. 2016) and is therefore not an ideal sterilisation method. Meanwhile, EtO and H_2_O_2_ provide effective sterilisation without thermal damage but require careful control to avoid chemical residues. Supercritical CO₂ sterilisation, performed at 120 bar and 40°C for 60 min, demonstrated high sterility with minimal material impact, positioning it as a promising method for microfluidic device sterilisation (Yavuz et al. 2016). Integrating inline sterilisation techniques, such as UV‐C sterilisation systems or closed‐loop designs with inline 0.2 µm filters, can reduce contamination risks during scaled‐up operation (Varma and Voldman 2018; Zhang et al. 2014). For instance, a microfluidic chip has been developed that is capable of sterilising aqueous samples at 120°C under 400 kPa pressure without boiling, highlighting innovative in‐chip sterilisation strategies (Zhang et al. 2014).

Future efforts must focus on standardising scalable and GMP‐compliant sterilisation protocols while maintaining device functionality and cost‐effectiveness. This includes packaging solutions like heat sealing or ultrasonic welding to prevent leakage and contamination, alongside real‐time contamination monitoring systems for improved quality assurance. These advancements will be essential to overcome the sterility barrier and enable large‐scale clinical EV production.

#### 5.1.6 Isolating Rare Functional EV Subpopulations at Scale

Addressing EV heterogeneity is critical for clinical translation. Emerging evidence suggests that only a small fraction of EVs from bulk cell populations exhibit the desired therapeutic phenotype (van de Wakker et al. 2023). Microfluidic systems are uniquely positioned to isolate such rare subpopulations. For example, Akbar et al. employed a herringbone microfluidic chip functionalised with anti‐VCAM‐1 antibodies to isolate rare VCAM‐1+ endothelial cell‐derived EVs from a heterogeneous population, demonstrating high capture specificity and enabling downstream detection of low‐abundance transcripts such as ICAM‐1 using ddPCR (Akbar et al. 2024).

Because only a small subset of EVs possesses the desired therapeutic phenotype, large‐scale isolation is essential but remains challenging for microfluidic platforms due to inherent throughput limitations. To address this, microfluidic devices have been successfully parallelised in formats such as immuno‐inertial and immunomagnetic platforms, enabling processing of up to litre‐scale samples within hours (see Section 3.2). One effective strategy involves combining size‐based pre‐enrichment modules with a downstream marker‐based capture step to enhance overall yield and selectivity. Such multimodal strategies not only compensate for low abundance but also enable consistent, scalable isolation of functionally relevant EVs with minimal sample loss.

Another strategy to address the challenge of isolating rare therapeutic EVs from bulk cell cultures is the generation of synthetic or semisynthetic EVs with defined characteristics. Microfluidic systems offer precise control over vesicle purification based on surface markers and cargo loading, making them well‐suited for this purpose (section 3.3). For a more focussed overview, Meng et al. (2021) comprehensively reviewed the use of microfluidic platforms for engineering EV mimetics, highlighting their potential for therapeutic delivery and the standardisation of EV manufacturing.

#### 5.1.7 Contamination Risks, Reusability and Purity of EVs in Microfluidic Platforms

Most current microfluidic EV production platforms are designed as single‐use systems, driven by the need to meet stringent GMP standards and prevent cross‐contamination between batches. Disposable cartridges and enclosed chips such as µPulse‐TFF (https://formulatrix.com/pulse‐tangential‐flow‐filtration/) and Exodus Bio are commonly used to minimise contamination risk, especially during EV handling and downstream purification steps. Closed‐loop microfluidic systems reduce manual intervention and exposure to external contaminants, which is particularly important in clinical workflows.

For large‐scale clinical‐grade EV manufacturing, integration with cleanroom environments or biosafety cabinets is often required to maintain sterility and product integrity throughout the production cycle. Although reusable chip designs are technically feasible, they would require robust, validated cleaning and sterilisation protocols to ensure compliance, standards that are still under development and not yet widely adopted.

Lastly, contaminants such as protein aggregates, cell debris and lipoproteins often overlap in size with sEVs (30–150 nm), making them difficult to remove using size‐based purification alone. Although microfluidic systems may not fully resolve this overlap on their own, hybrid strategies that combine size‐based separation with affinity capture, inertial enrichment or magnetic bead‐based isolation can provide enhanced selectivity.

### 5.2 Potential Impact of Microfluidics on Commercialisation and Clinical Translation

Microfluidic technologies are poised to revolutionise the commercialisation and clinical translation of EV‐based therapies by addressing cost, efficiency and scalability challenges associated with traditional EV production and purification methods. Compared to UC, microfluidic systems reduce manufacturing costs significantly; for instance, microfluidic‐based purification reduces reagent volumes, elimination of bulky equipment and automates workflows (Chen et al. 2023; De Sousa et al. 2023). Microfluidic bioreactors can further improve production throughput, with systems scaling capacity from 10¹⁰ EVs/h to upwards of 10¹^2^ EVs/h while maintaining consistent purity and therapeutic efficacy.

The operational costs of microfluidic devices are minimised due to the low consumption of materials, such as antibodies and filters, as compared to batch‐based approaches like TFF or density‐gradient UC (Dilsiz 2024). Moreover, microfluidic platforms integrated with inline QC tools, such as optical sensors and impedance‐based monitoring systems, streamline GMP compliance, reducing time and costs associated with off‐line characterisation (Meng et al. 2021; Kang and Liu 2024). For example, a closed‐loop microfluidic system incorporating inline monitoring can provide real‐time analysis of EV yield, size and purity with less than 5% variability between batches (De Sousa et al. 2023).

Despite these advantages, challenges remain for scaling microfluidic systems to meet clinical demand, particularly in ensuring sterility and overcoming fabrication costs for single‐use devices. Advances in scalable manufacturing techniques, such as injection moulding of thermoplastics and integration of UV‐C sterilisation modules, are expected to enhance the clinical viability of microfluidic systems (Meng et al. 2021; Kang and Liu 2024). With continued optimisation, microfluidics holds immense potential to reduce production costs, accelerate EV manufacturing workflows and ensure the quality and consistency required for large‐scale clinical applications.

### 5.3 Emerging Trends and Innovations

Several key innovations in microfluidics promise to overcome technical limitations, streamline manufacturing, and enable advanced EV applications:

#### 5.3.1 AI Integration and Automation

The integration of artificial intelligence (AI) with microfluidic systems represents a transformative innovation in EV manufacture, particularly in production optimisation, purification, characterisation, and QC. AI algorithms enable real‐time data acquisition and analysis, ensuring precise monitoring and control of EV size, molecular cargo, and production yields​ (Chen et al. 2023; Greenberg et al. 2023). Machine learning models can predict and adjust flow rates, shear stress, and filtration parameters to maintain consistent EV size distribution and bioactivity across production batches (Greenberg et al. 2023). This automation eliminates variability often observed in manual or static methods and accelerates EV manufacturing for clinical use.

AI further enhances the downstream purification and characterisation stages of EV production. By analysing complex datasets, AI models can quickly assess EV molecular signatures, detect impurities, and optimise isolation parameters. For example, deep learning algorithms applied to nanoparticle tracking data have been shown to identify subtle changes in EV size and purity that traditional methods may overlook, ensuring stringent QC (Liu et al. 2021). Additionally, automated AI systems integrated with inline sensors in microfluidic devices can detect anomalies such as flow disruptions or contamination in real time, triggering corrective actions to maintain process reliability. At the analytical stage, AI‐powered tools streamline the interpretation of high‐throughput omics data obtained during EV characterisation, enabling rapid identification of biomarkers or functional cargo (Chen et al. 2023)​. Such automation improves the reproducibility of EV products for clinical applications and ensures compliance with GMP standards.

Future advancements will focus on combining AI with feedback‐loop control systems to dynamically tune microfluidic device performance. This includes optimising parameters such as channel geometry, fluid dynamics, and sensor outputs to ensure maximum EV yield, purity, and therapeutic efficacy (Shah et al. 2024)​. With AI‐driven automation, microfluidic systems can evolve into fully integrated platforms capable of producing high‐quality EVs at scale, facilitating their commercialisation and clinical translation.

#### 5.3.2 Personalised Manufacturing

Microfluidics allows on‐demand and localised EV production, which is particularly advantageous for clinical settings where rapid, tailored production is necessary. Systems like droplet microfluidics enable encapsulation and collection of EVs from photodegradable hydrogels, achieving efficient, on‐demand EV delivery in under 2 min upon UV stimulation (Kim et al. 2023). This ensures precise delivery of EVs while preserving their integrity, a critical step for applications such as wound healing and localised drug delivery.

Personalised EV manufacture can also be facilitated through microfluidic‐based analysis of a patient's EVs. By performing rapid molecular profiling of EVs isolated from body fluids using microfluidics, personalised therapies can be designed based on the molecular signature of the patient's disease state (Contreras‐Naranjo et al. 2017; Kang et al. 2020). For instance, the extracellular vesicle‐on‐demand (*EVOD*) chip enables high‐specificity isolation and real‐time quantification of cancer‐associated EVs using immunoaffinity methods, aiding in patient‐specific diagnostics and treatment monitoring (Kang et al. 2020)​.

Furthermore, integrating organ‐on‐chip platforms with microfluidics represents a powerful approach for personalised medicine. Patient‐derived cells and tissues can be cultured on organ‐on‐chip systems to evaluate the therapeutic efficacy of EVs in real‐time, mimicking physiological environments. Digital organ‐on‐chip systems have been developed to test the efficacy of EV‐based therapies, such as natural killer cell‐derived EVs, on liver cancer​ (Wu et al. 2022). These platforms allow parallelised, high‐throughput analysis of therapeutic responses, providing insights into individualised treatment strategies.

Emerging trends also include microfluidic‐based engineering of EVs for on‐demand drug delivery. Surface‐engineered EVs can be functionalised with therapeutic cargo, such as tumour antigens for immunotherapy, using microfluidic systems that streamline isolation, modification and release in a single workflow (Zhao et al. 2019; Piffoux et al. 2017). This minimises processing times and improves precision, offering potential for real‐time, patient‐specific EV therapies.

### 5.4 Bridging Microfluidic Manufacturing and Therapeutic Translation

#### 5.4.1 Therapeutic Applications Enabled by Microfluidic EV Manufacturing

Microfluidic technologies have transcended their role in EV isolation and purification, directly contributing to therapeutic advancements across various disease models. Although full adoption of microfluidic technology in clinical‐grade EV therapeutics is still at its infancy, we highlight specific instances where microfluidic‐manufactured EVs have demonstrated tangible preclinical and clinical benefits:

- *Shear‐optimised bioreactor‐derived MSC‐EVs promote vascular regeneration in diabetic wounds*: A microfluidic shear‐optimised bioreactor has been employed to produce MSC‐EVs with superior regenerative capabilities (Kronstadt et al. 2023). In diabetic mouse models, these EVs significantly accelerated wound closure and promoted angiogenesis, outperforming EVs produced via conventional methods.
- *GelMA‐integrated microfluidic bioreactor enhances EV yield and healing efficacy in diabetic ulcers*: A herringbone‐structured microfluidic bioreactor with integrated GelMA microcarriers enabled the scalable production of bone marrow‐derived MSC‐EVs (Huang et al. 2023). These EVs, when subcutaneously injected into diabetic rats with dorsal wounds, significantly accelerated wound closure, enhanced angiogenesis, promoted collagen deposition and suppressed inflammation, achieving over 80% wound closure by Day 14‐matching or exceeding static‐culture‐derived EVs.
- *Microfluidic 3D model validates cartilage penetration of therapeutic EVs*: In the ongoing GelVex clinical trial (NCT06713902) (I.R.C.C.S Ospedale Galeazzi‐Sant'Ambrogio 2024), MSC‐EVs are incorporated into a platelet‐rich plasma‐based fibrin gel for cartilage repair in osteoarthritis patients. A 3D microfluidic model is employed to assess the EVs’ ability to penetrate cartilage and to be internalised by human chondrocytes, offering a physiologically relevant platform for preclinical validation.
- *3D organ‐on‐chip models reveal functional impact of adipose‐derived EVs in metabolic disease*: In the EVOC clinical trial (NCT06408961) (Das 2024), EVs isolated from subcutaneous and visceral fat tissues of patients undergoing bariatric surgery are used to treat 3D liver‐on‐chip and cardiomyocyte models. This platform enables direct evaluation of how fat‐derived EV cargo influences cardiac and hepatic function, including gene expression and electrophysiological changes. It exemplifies how microfluidic organ‐on‐chip systems can bridge molecular EV profiling with functional validation, advancing therapeutic insights in cardiometabolic disease.
- *Microfluidic stimulation boosts EV yield and accelerates corneal wound healing*: Using a custom‐designed microfluidic device called *SEED* (Hao et al. 2023), researchers mechanically stimulated MSCs to significantly increase the secretion of EVs, by approximately four‐fold, without compromising cell viability or EV function. These EVs retained their regenerative potential and, when applied to human corneal epithelial cells in a wound healing model, accelerated scratch closure and upregulated key repair‐related genes (e.g., IL‐6, TGF‐β1 and ZO‐1). This study demonstrates how microfluidic mechanical stimulation can effectively enhance EV yield while preserving or enhancing therapeutic bioactivity in clinically relevant tissue models.
- *Microfluidic loading enhances EV‐based chemotherapy for glioma*: A custom‐designed microfluidic device (Exo‐Load) (Thakur et al. 2020) was developed to improve the loading of doxorubicin into exosomes derived from glioma cells. Compared to conventional loading methods, this approach significantly increased drug encapsulation efficiency, particularly when using a sigmoid channel design. The loaded EVs showed stronger uptake by autologous glioma cells and led to a greater reduction in cell proliferation than free drug alone. These findings suggest that microfluidic platforms could play a valuable role in refining EV‐based drug delivery for brain tumours like glioma.
- *Microfluidic‐engineered hybrid vesicles improve in vivo gene delivery*: In a preclinical study, researchers used microfluidic mixing to generate hybrid nanoparticles by fusing plasma membrane‐derived vesicles with LNPs, enhancing both structure and function (Alter et al. 2025). Compared to standard LNPs, these hybrids delivered significantly more mRNA in vitro and in vivo, with up to 18‐fold higher gene expression observed in zebrafish larvae and mice. The enhanced therapeutic performance was linked to improved endosomal escape and more efficient intracellular trafficking, highlighting the translational potential of microfluidic manufacturing for next‐generation gene delivery systems.
- *Formulatrix‐based TFF enables potent MSC secretome therapy for diabetic peripheral artery disease*: A TFF system incorporating Formulatrix membranes with a 10–300 kDa molecular weight cutoff was used to isolate the secretome from hypoxia‐preconditioned MSCs (Sazli et al. 2023). This size range likely retains EVs, including exosomes, along with soluble cytokines. The resulting secretome (S‐MSCs) was enriched with proangiogenic and antiinflammatory factors such as VEGF, PDGF, bFGF, IL‐10 and TGF‐β. In a diabetic rat model of peripheral artery disease, intramuscular injection of S‐MSCs improved limb motor function, increased VEGF gene expression and enhanced CD31+ vascular density.

#### 5.4.2 Pathways for Clinical Integration of Microfluidic EV Platforms

Translating microfluidic EV technologies from proof‐of‐concept to clinical‐grade manufacturing requires alignment with the operational, regulatory and logistical frameworks of therapeutic development. As recently outlined in broader reviews on EV‐based therapeutics and GMP translation (Estes et al. 2022; Grangier et al. 2021), key challenges include standardisation, scalability and compliance with quality frameworks. One practical direction is the development of modular, single‐use cartridges that can be integrated into existing GMP infrastructure. These self‐contained systems could minimise contamination risk while enabling closed‐loop EV production directly from clinical cell sources, such as patient‐derived MSCs or immune cells. Another promising path is the application of microfluidic systems in autologous workflows (Moskovitch et al. 2025), where EVs are isolated and processed from a patient's own cells and reinfused. This approach may lower regulatory hurdles in early‐stage clinical trials.

Additionally, to support broader adoption, device manufacturers and EV developers will need to co‐develop platforms with harmonised process controls, validated release criteria and flexible interfaces for upstream and downstream modules (Sharma et al. 2024; Marques and Szita 2017). Regulatory agencies are increasingly open to platform‐based submissions, especially for cell and gene therapies; thus, the adoption of microfluidic EV systems could benefit from early engagement with regulatory bodies. In parallel, hybrid vesicle technologies that combine EVs with synthetic carriers (Zhang et al. 2023; Alter et al. 2025; Pareja Tello et al. 2025), such as LNPs, may offer an accessible clinical entry point, leveraging the regulatory familiarity and delivery efficiency of existing LNP platforms while preserving the targeting and cargo advantages of EVs.

### 5.5 Regulatory Status of EVs, Current Regulatory Framework and Path Forward

EV‐based therapeutics are making significant progress with growing interest in their potential applications across diverse therapeutic areas. As of now, of the 471 EV‐related clinical trials in more than 200 conditions, only 19.32% involve EV‐based therapeutics (Mizenko et al. 2024). The majority of EVs used in these trials are native EVs, and 15% use engineered EVs. A small portion of these trials have recently advanced to Phase 3, reflecting the promise of EV‐based therapeutics. Despite these encouraging outcomes the following key challenges and considerations need to be addressed:

1. A harmonised regulatory framework. Regulatory authorities, including the FDA (USA), EMA (Europe) and TGA (Australia), are gradually addressing the unique challenges of EV‐based therapies, including acceptable standards for size distribution ranges, particle‐to‐protein ratios and criteria for contaminant removal. Despite progress, the absence of consistent globally harmonised guidelines complicates uniform production practices, creating variability across manufacturers.
2. Industry standards for QC and microfluidic systems. Establishing robust standards for EV production is critical for achieving consistent therapeutic outcomes. Emerging benchmarks include size distribution ranges of 50–150 nm; protein content thresholds such as <1 µg of protein per 10^9^ EVs. However, widespread adoption remains uneven due to variability in EV manufacturing processes and analytical methods among producers. Microfluidic technologies, with their precision control, capacity for inline QC, ability to produce EVs with reproducible characteristics, are uniquely positioned to meet emerging regulatory standards. As these technologies advance, they will play a crucial role in achieving compliance with future guidelines.
3. Path forward. To unlock the full therapeutic potential of EVs, collaboration between the EV scientific community, industry stakeholders and regulatory authorities is essential. Joint efforts can establish enforceable standards that ensure: (i) uniformity in EV manufacturing, isolation and characterisation, (ii) safety and efficacy across different therapeutic applications and (iii) streamlined regulatory submissions and approvals.

Advances in microfluidic technology and bioanalytics are improving the scalability, reproducibility and QC of EV production. As interest in EV‐based therapeutics grows, regulatory authorities are gradually creating guidelines tailored to EV‐based therapeutics, laying a foundation for potential approvals. The approval of the first EV‐based therapeutic will set a critical precedent, catalysing the field and paving the way for subsequent approvals. Aligning innovation and emerging regulations will be pivotal for the clinical and commercial success of the EV field. The development and integration of microfluidic‐based technologies for EV analytics and manufacture is required to underpin the advance of EV therapeutics to the clinic. Further collaboration between academia, industry and regulatory bodies will be essential to establish robust, enforceable standards and unlock the full therapeutic potential of EVs.

## Author Contributions

**Amin Hassanzadehbarforoushi**: Conceptualization (equal); data curation (equal); investigation (equal); methodology (equal); writing–original draft (equal); writing–review and editing (equal). **Xenia Sango**: Writing ‐ original draft (equal); writing ‐ review and editing (equal). **Ella Johnston**: Writing–review and editing (equal). **David Haylock**: Funding acquisition (equal); project administration (supporting); writing ‐ original draft (equal); writing ‐ review and editing (equal). **Yuling Wang**: Conceptualization (equal); funding acquisition (equal); project administration (equal); supervision (equal); writing–review and editing (equal).

## Conflicts of Interest

The authors declare no conflicts of interest.

## Data Availability

The data that support the findings of this study are available from the corresponding author upon reasonable request.
